# Supplementary material for: The Bor1 elevator transport cycle is subject to autoinhibition and activation
Source: Nat Commun. 2024 Oct 22;15:9090. doi: 10.1038/s41467-024-53411-1 (PMC11494103; doi:10.1038/s41467-024-53411-1)
Supplement: Supplementary file 1 — Supplementary Information [file 41467_2024_53411_MOESM1_ESM.pdf]

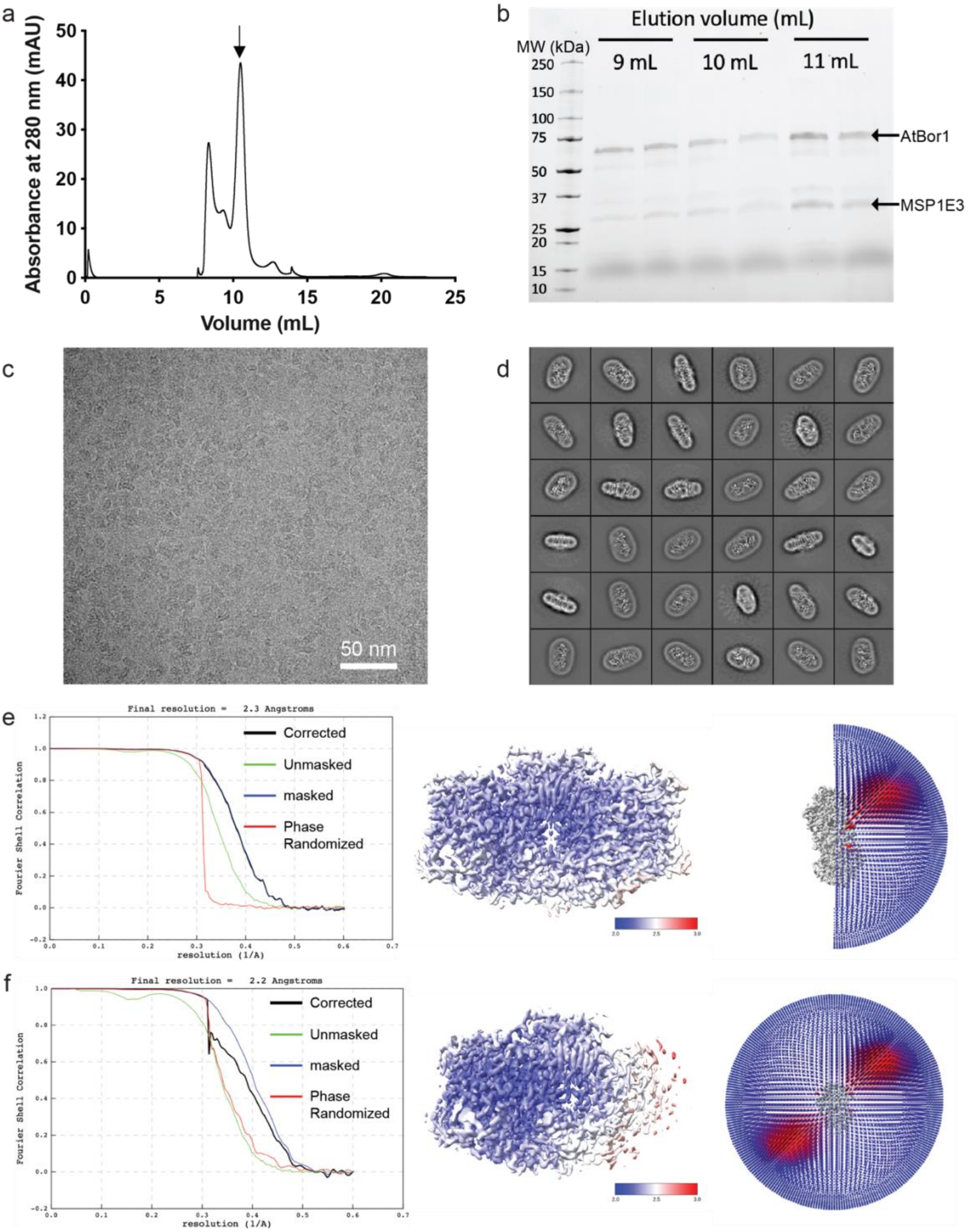
a
Absorbance at 280 nm (mAU)
Volume (mL)
b
Elution volume (mL)
MW (kDa)
9 mL
10 mL
11 mL
AtBor1
MSP1E3
c
50 nm
d
e
Final resolution = 2.3 Angstroms
Fourier Shell Correlation
resolution (1/A)
Corrected
Unmasked
masked
Phase Randomized
f
Final resolution = 2.2 Angstroms
Fourier Shell Correlation
resolution (1/A)
Corrected
Unmasked
masked
Phase Randomized

Supplementary Fig. 1 **Cryo-EM analysis of wild-type AtBor1 in lipid nanodiscs. a** Chromatographs of wild-type AtBOR1 in lipid nanodiscs. The arrowed peak was collected for cryo-EM study. **b** The SDS-PAGE of the peak fractions. Monomeric AtBor1 protein was observed clearly on the SDS-PAGE between 75 to 50 KDa and the MSP1E3 protein was observed on the SDS-PAGE at ~25KDa. **c** A representative electron micrograph of wild-type AtBor1 in lipid nanodiscs. **d** Representative two-dimensional class averages of the electron micrographs for wild-type AtBor1 in lipid nanodiscs. **e, f** Fourier Shell Correlation (FSC) curves (left), local resolution maps (middle), and particle orientation plots (right) of the 3D reconstruction of inward-autoinhibition dimer (**e**) and inward-autoinhibition protomer (**f**).

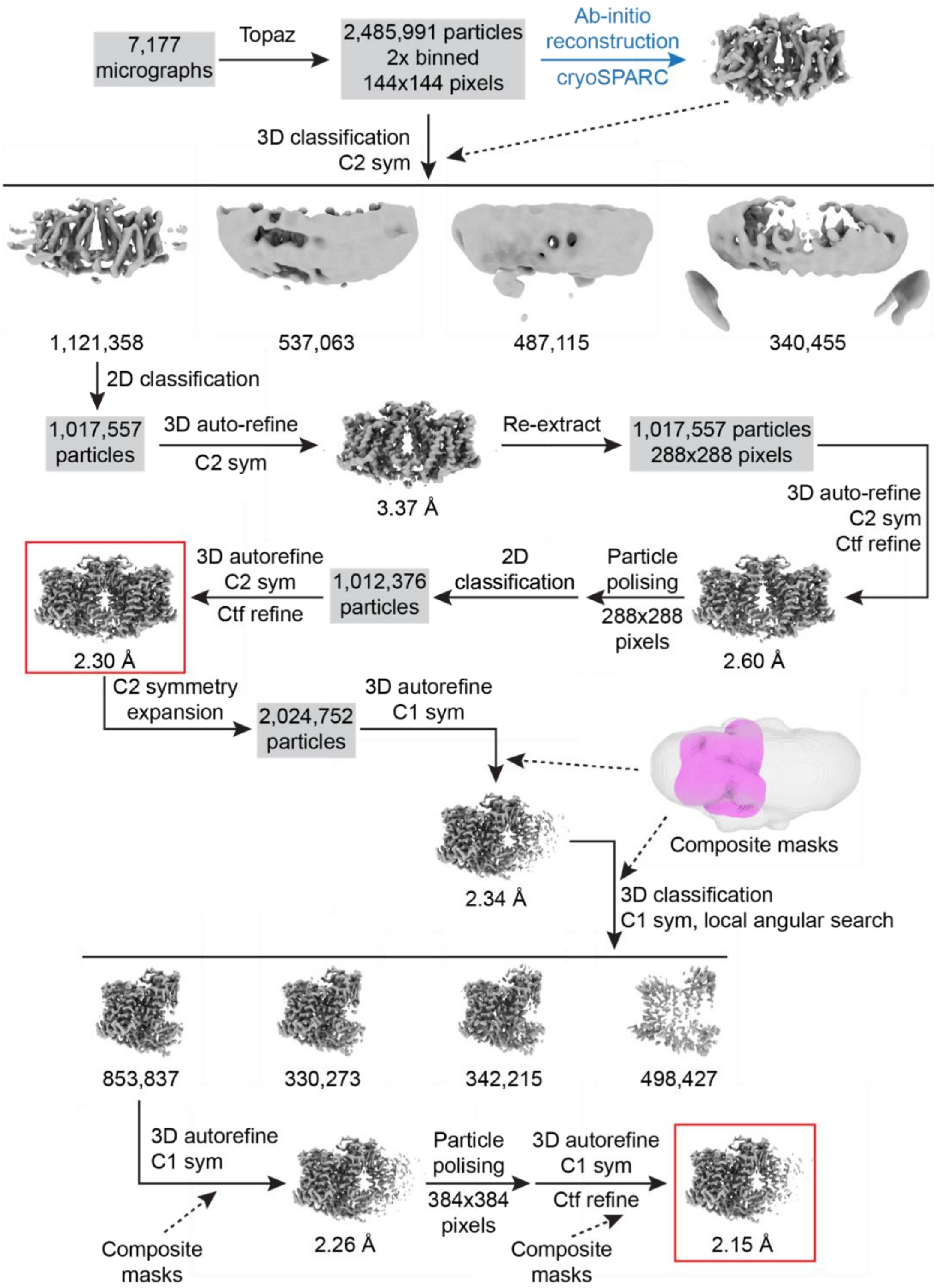

Supplementary Fig. 2 **Cryo-EM data processing workflow for wild-type AtBor1**. The 3D reconstructions are shown without B-factor sharpening. The reconstructions in red boxes are used for atomic model building.

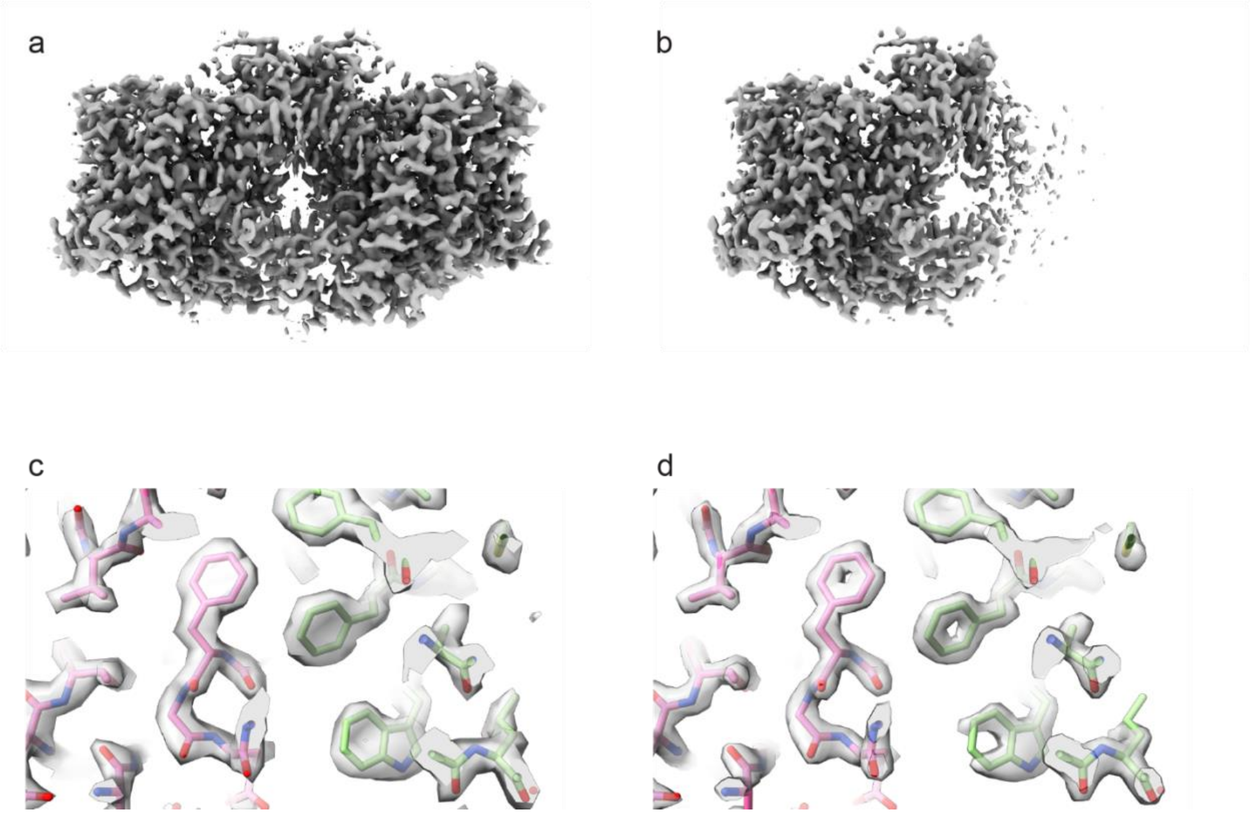

Supplementary Fig. 3 **Comparison of the cryo-EM density maps of wild-type AtBor1 dimer and protomer. a, b** B-factor sharpened map of wild-type AtBor1 dimer (**a**) or protomer (**b**). **c, d** Close-up view of the same region in the density map of wild-type AtBor1 dimer (**c**) or protomer (**d**). The holes in some aromatic residues are clearly resolved in (**d**), suggesting an improved resolution.

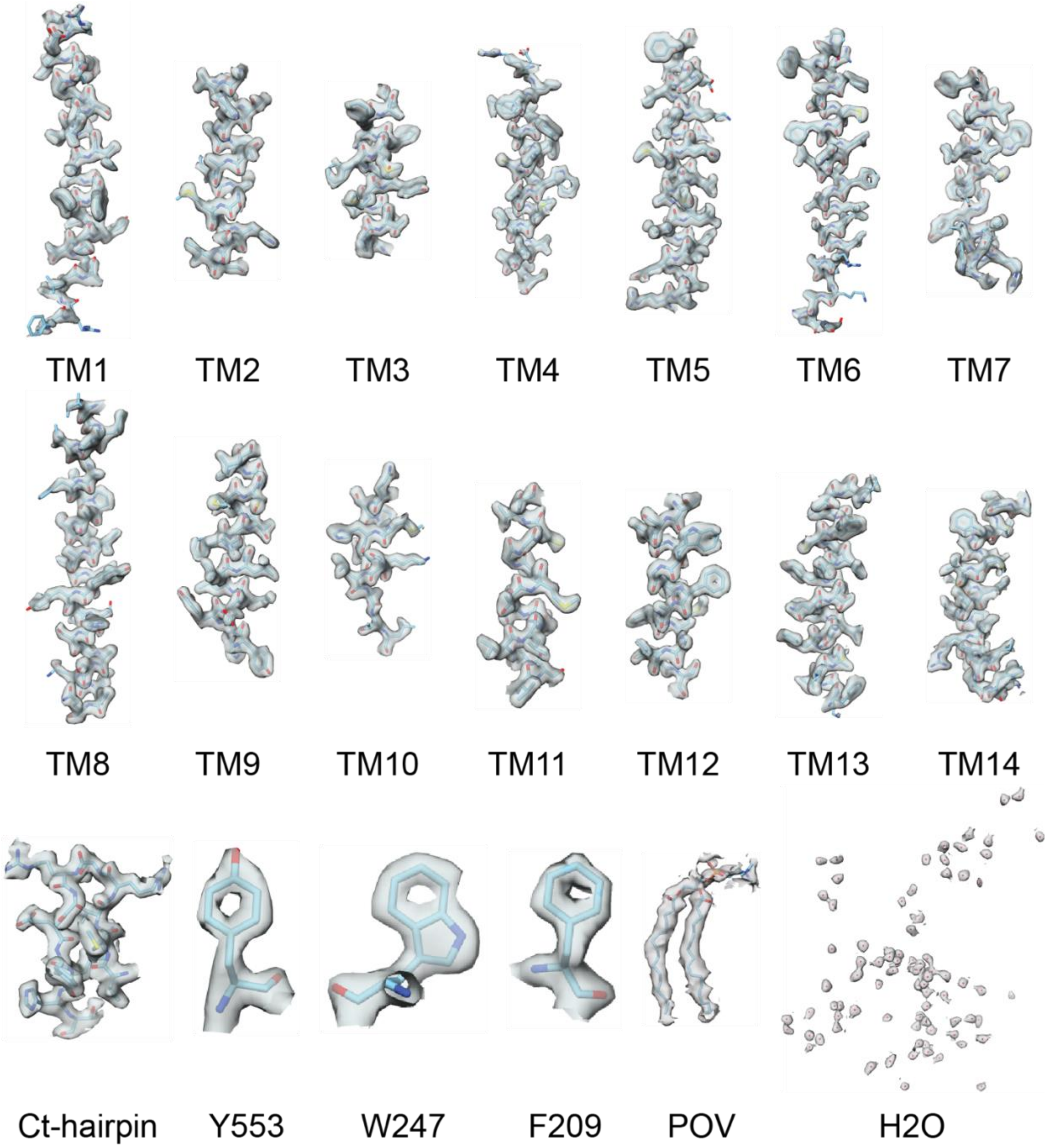

Supplementary Fig. 4 **Cryo-EM maps for representative regions of wild-type AtBor1**. The cryo-EM maps for the TM1-TM14 helices, the Ct-hairpin region, the selected tyrosine (Y), tryptophan (W) and phenylalanine (F) residues, the POPC (POV) lipid and the water molecules of wild-type AtBor1.

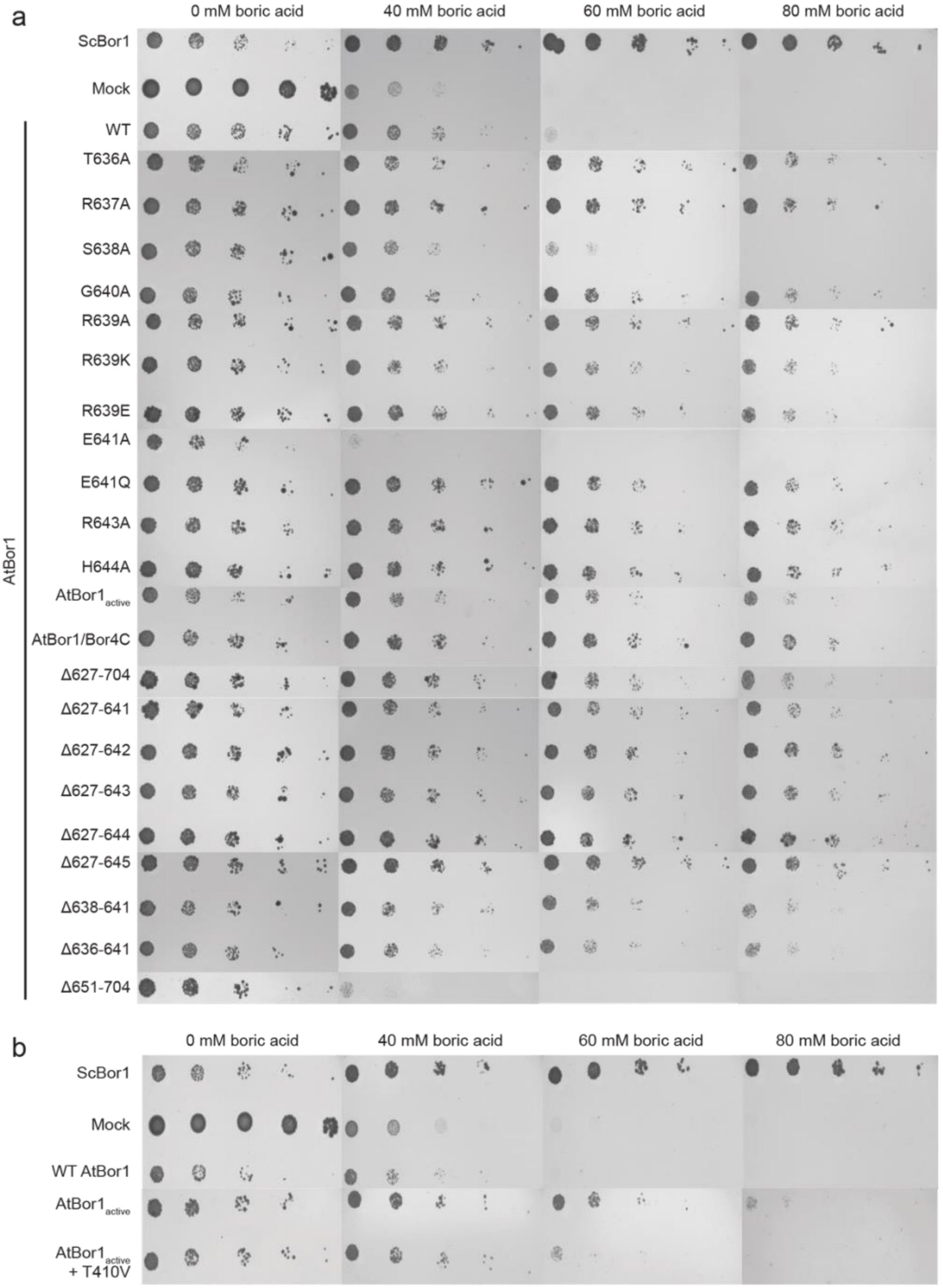

Supplementary Fig. 5 **Complementation assay identifies the Ct hairpin inhibited the transporter activity of AtBor1. a, b** *S. cerevisiae* strain Y01169 (*Scbor1Δ*) were transformed with the Ct domain truncated or mutant AtBor1 cDNAs. Overnight liquid cultures of the transformants were adjust to an OD_{600nm} of 1.0 and serially diluted by 5 times, spotted on solid media containing 0 mM (control), 40 mM, 60 mM and 80 mM boric acid and incubated for 6 days. Galactose was used as carbon source to induce the expression of the AtBor1s.

```
AtBor1|Q8VYR7        -------------------------------------------------------------------------------- 0
AtBor2|Q9M1P7        -------------------------------------------------------------------------------- 0
AtBor3|Q93Z13        -------------------------------------------------------------------------------- 0
AtBor4|Q9XI23        -------------------------------------------------------------------------------- 0
AtBor5|Q9SSG5        -------------------------------------------------------------------------------- 0
AtBor6|Q3E954        -------------------------------------------------------------------------------- 0
AtBor7|Q9SUU1        -------------------------------------------------------------------------------- 0
OsBor1|Q2QNH0        -------------------------------------------------------------------------------- 0
OsBor2|Q1ZYR7        -------------------------------------------------------------------------------- 0
OsBor3|Q7X9F3        -------------------------------------------------------------------------------- 0
OsBor4|Q1ZYR6        -------------------------------------------------------------------------------- 0
TaBor1.1|A0A060PT44  -------------------------------------------------------------------------------- 0
TaBor1.2|A0A060PVQ3  -------------------------------------------------------------------------------- 0
TaBor1.3|W5FNG8      -------------------------------------------------------------------------------- 0
ScBor1|P53838        -------------------------------------------------------------------------------- 0
SLC4A11|Q8NBS3       -------------------------------------------------------------------------------- 0
SLC4A1/AE1|P02730    -------------------------------------------------------------------------------- 0
SLC4A2/AE2|P04920    MSSAPRRPAKGADSFCTPEPESLGPGTPGFPEQEEDELHRTLGVERFEEILQEAGSRGGEEPGRSYGEEDFEYHRQSSHH 80
SLC4A3/AE3|P48751    MANGVIPPPGGASPLPQVRVPLEEPPLSPDVEEEDDDLGKTLAVSRFGDLISKPPAWDPEKPSRSYSERDFEFHRHTSHH 80
SLC4A4/NBCe1|Q9Y6R1  -------------------------------------------------------------------------------- 0
SLC4A5/NBCe2|Q9BY07  -------------------------------------------------------------------------------- 0
SLC4A7/NBCn1|Q9Y6M7  -------------------------------------------------------------------------------- 0
SLC4A8/NDCBE|Q2Y0W8  -------------------------------------------------------------------------------- 0
SLC4A9/AE4|Q96Q91    -------------------------------------------------------------------------------- 0
SLC4A10/NBCn2|Q6U841 -------------------------------------------------------------------------------- 0

AtBor1|Q8VYR7        -------------------------------------------------------------------------------- 0
AtBor2|Q9M1P7        -------------------------------------------------------------------------------- 0
AtBor3|Q93Z13        -------------------------------------------------------------------------------- 0
AtBor4|Q9XI23        -------------------------------------------------------------------------------- 0
AtBor5|Q9SSG5        -------------------------------------------------------------------------------- 0
AtBor6|Q3E954        -------------------------------------------------------------------------------- 0
AtBor7|Q9SUU1        -------------------------------------------------------------------------------- 0
OsBor1|Q2QNH0        -------------------------------------------------------------------------------- 0
OsBor2|Q1ZYR7        -------------------------------------------------------------------------------- 0
OsBor3|Q7X9F3        -------------------------------------------------------------------------------- 0
OsBor4|Q1ZYR6        -------------------------------------------------------------------------------- 0
TaBor1.1|A0A060PT44  -------------------------------------------------------------------------------- 0
TaBor1.2|A0A060PVQ3  -------------------------------------------------------------------------------- 0
TaBor1.3|W5FNG8      -------------------------------------------------------------------------------- 0
ScBor1|P53838        -------------------------------------------------------------------------------- 0
SLC4A11|Q8NBS3       -------------------------------------------------------------------------------- 0
SLC4A1/AE1|P02730    -------------------------------------------------------------------------------- 0
SLC4A2/AE2|P04920    IHHPLSTHLPPDARRRKTPQGPGRKPRRRPG-----ASPTGETPTI-EEGEEDED------EASEAEGARALTQPSPVST 148
SLC4A3/AE3|P48751    THHPLSARLPPPHKLRRLPPTSARHTRRKRKKEKTSAPPSEGTPPIQEEGGAGVDEEEEEEEEEEGESEAEPVEPPHSGT 160
SLC4A4/NBCe1|Q9Y6R1  -------------------------------------------------------------------------------- 0
SLC4A5/NBCe2|Q9BY07  -------------------------------------------------------------------------------- 0
SLC4A7/NBCn1|Q9Y6M7  -------------------------------------------------------------------------------- 0
SLC4A8/NDCBE|Q2Y0W8  -------------------------------------------------------------------------------- 0
SLC4A9/AE4|Q96Q91    -------------------------------------------------------------------------------- 0
SLC4A10/NBCn2|Q6U841 -------------------------------------------------------------------------------- 0

AtBor1|Q8VYR7        -------------------------------------------------------------------------------- 0
AtBor2|Q9M1P7        -------------------------------------------------------------------------------- 0
AtBor3|Q93Z13        -------------------------------------------------------------------------------- 0
AtBor4|Q9XI23        -------------------------------------------------------------------------------- 0
AtBor5|Q9SSG5        -------------------------------------------------------------------------------- 0
AtBor6|Q3E954        -------------------------------------------------------------------------------- 0
AtBor7|Q9SUU1        -------------------------------------------------------------------------------- 0
OsBor1|Q2QNH0        -------------------------------------------------------------------------------- 0
OsBor2|Q1ZYR7        -------------------------------------------------------------------------------- 0
OsBor3|Q7X9F3        -------------------------------------------------------------------------------- 0
OsBor4|Q1ZYR6        -------------------------------------------------------------------------------- 0
TaBor1.1|A0A060PT44  -------------------------------------------------------------------------------- 0
TaBor1.2|A0A060PVQ3  -------------------------------------------------------------------------------- 0
TaBor1.3|W5FNG8      -------------------------------------------------------------------------------- 0
ScBor1|P53838        -------------------------------------------------------------------------------- 0
SLC4A11|Q8NBS3       -------------------------------------------------------------------------------- 0
SLC4A1/AE1|P02730    -------------------------------------------------------------------------------- 0
SLC4A2/AE2|P04920    PSSVQFFLQEDDSADRK-AERTSPSS--PAPLPHQEATPRASK--GAQAGTQVEEAEAEAVAVASGTAGGDDGGASGRPL 223
SLC4A3/AE3|P48751    PQKAKFSIGSDEDDSPGLPGRAAVTKPLPSVGPHTDKSPQHSS--SS------PSPR----ARAS-----RLAGEKSRP- 222
SLC4A4/NBCe1|Q9Y6R1  --------------------------------MEDEAVLDRGASFLKHVCD---EEEVEG----------HHTIYIGVHV 35
SLC4A5/NBCe2|Q9BY07  --------------------------------MKVK-EEKAGVGKLDHTNHRRRFPDQKE----------CPPIHIGLPV 37
SLC4A7/NBCn1|Q9Y6M7  ---------------ME-----RFRLEKKLPGPDEEAVVDLGKT-SSTVNTKFEKEELES----------HRAVYIGVHV 49
SLC4A8/NDCBE|Q2Y0W8  ---------------MPAAGSNEPDGVLSYQRPDEEAVVDQGGT-STILNIHYEKEELEG----------HRTLYVGVRM 54
SLC4A9/AE4|Q96Q91    --------------------------------------------------MEMKLPGQEG----------FEAS------ 14
SLC4A10/NBCn2|Q6U841 ---------------MEIKDQGAQMEPLLPTRNDEEAVVDRGGT-RSILKTHFEKEDLEG----------HRTLFIGVHV 54
```

```
AtBor1|Q8VYR7               -------------------------------------------------------------------------------- 0
AtBor2|Q9M1P7               -------------------------------------------------------------------------------- 0
AtBor3|Q93Z13               -------------------------------------------------------------------------------- 0
AtBor4|Q9XI23               -------------------------------------------------------------------------------- 0
AtBor5|Q9SSG5               -------------------------------------------------------------------------------- 0
AtBor6|Q3E954               -------------------------------------------------------------------------------- 0
AtBor7|Q9SUU1               -------------------------------------------------------------------------------- 0
OsBor1|Q2QNH0               -------------------------------------------------------------------------------- 0
OsBor2|Q1ZYR7               -------------------------------------------------------------------------------- 0
OsBor3|Q7X9F3               -------------------------------------------------------------------------------- 0
OsBor4|Q1ZYR6               -------------------------------------------------------------------------------- 0
TaBor1.1|A0A060PT44         -------------------------------------------------------------------------------- 0
TaBor1.2|A0A060PVQ3         -------------------------------------------------------------------------------- 0
TaBor1.3|W5FNG8             -------------------------------------------------------------------------------- 0
ScBor1|P53838               -------------------------------------------------------------------------------- 0
SLC4A11|Q8NBS3              --------------------MAAATRRVFHL---QPCEN----------SPTMSQNGYFEDSSYYKCDT----------- 36
SLC4A1/AE1|P02730           ------------------------------------MEE------LQDDYEDM-MEENLEQEEYEDPDIPESQMEEPAAH 37
SLC4A2/AE2|P04920           PKAQPGHRSYNLQERRRIGSMTGA---EQALLPRVPTDEIEAQTLATADLDLM-KSHRFED----VPGVRRHLVRKNAKG 295
SLC4A3/AE3|P48751           ---WSPSASYDLRERLCPGSALGN---PGGPEQQVPTDEAEAQMLGSADLDDM-KSHRLED----NPGVRRHLVKKPSRT 291
SLC4A4/NBCe1|Q9Y6R1         PKSYR--RR--RRHKRKTGHKEKKEKE-------RISEN------------------------YSDKSDIE------NAD 74
SLC4A5/NBCe2|Q9BY07         PTYPQ--RK--TDQK---GHLSGLQKVHWGLRPDQPQQE------------------------LTGPGSGA------SSQ 80
SLC4A7/NBCn1|Q9Y6M7         PFS-K--ES--RRRHRHRGHKHHHRRRKD------------------------------------KESDKE--------- 79
SLC4A8/NDCBE|Q2Y0W8         PLG-R--QS--HRHHRTHGQKHRRRGRGK------------------------------------GASQGE--------- 84
SLC4A9/AE4|Q96Q91           --------S--APRNIPSGELDSNPDPGT--------------------------------------------------- 33
SLC4A10/NBCn2|Q6U841        PLGGR--KS--HRRHRHRGHKHRKRDRER------------------------------------D-SGLE--------- 84

AtBor1|Q8VYR7               -------------------------------------------------------------------------------- 0
AtBor2|Q9M1P7               -------------------------------------------------------------------------------- 0
AtBor3|Q93Z13               -------------------------------------------------------------------------------- 0
AtBor4|Q9XI23               -------------------------------------------------------------------------------- 0
AtBor5|Q9SSG5               -------------------------------------------------------------------------------- 0
AtBor6|Q3E954               -------------------------------------------------------------------------------- 0
AtBor7|Q9SUU1               -------------------------------------------------------------------------------- 0
OsBor1|Q2QNH0               -------------------------------------------------------------------------------- 0
OsBor2|Q1ZYR7               -------------------------------------------------------------------------------- 0
OsBor3|Q7X9F3               -------------------------------------------------------------------------------- 0
OsBor4|Q1ZYR6               -------------------------------------------------------------------------------- 0
TaBor1.1|A0A060PT44         -------------------------------------------------------------------------------- 0
TaBor1.2|A0A060PVQ3         -------------------------------------------------------------------------------- 0
TaBor1.3|W5FNG8             -------------------------------------------------------------------------------- 0
ScBor1|P53838               -------------------------------------------------------------------------------- 0
SLC4A11|Q8NBS3              -----------DDTFEAREEILGDEAFDTANSSIVSGESIRFFVNVNLEMQA-TNTENEATS----GG------------ 88
SLC4A1/AE1|P02730           DTE----------------ATATDYHTTSHP------GTHKVYVELQELVMDEKNQELRWME------------------ 77
SLC4A2/AE2|P04920           STQSGREGRE-PGPTPR-------ARPRAPH------KPHEVFVELNELLLD-KNQEPQWRE------------------ 342
SLC4A3/AE3|P48751           QGG---RGSP-SGLAPI--LRRKKKKKKLDR------RPHEVFVELNELMLD-RSQEPHWRE------------------ 340
SLC4A4/NBCe1|Q9Y6R1         ESSSSILKPLISPAAERIRFILGEED--DSP------APPQLFTELDELLAV-DGQEMEWKE------------------ 127
SLC4A5/NBCe2|Q9BY07         DSSMDLISRTRSPAAEQLQDILGEED--EAP------NP-TLFTEMDT-LQH-DGDQMEWKE------------------ 131
SLC4A7/NBCn1|Q9Y6M7         ---DGRESPSYDTPSQRVQFILGTEDDDEEH------IPHDLFTEMDELCYR-DGEEYEWKE------------------ 131
SLC4A8/NDCBE|Q2Y0W8         ---EGLEALAHDTPSQRVQFILGTEE-DEEH------VPHELFTELDEICMK-EGEDAEWKE------------------ 135
SLC4A9/AE4|Q96Q91           -------GPSPDGPSDTESKELG------VP------KDPLLFIQLNELLG--WPQALEWRETGSSSASLLLDMGEMPSI 92
SLC4A10/NBCn2|Q6U841        ---DGRESPSFDTPSQRVQFILGTEDDDEEH------IPHDLFTELDEICWR-EGEDAEWRE------------------ 136

AtBor1|Q8VYR7               -------------------------------------------------------------------------------- 0
AtBor2|Q9M1P7               -------------------------------------------------------------------------------- 0
AtBor3|Q93Z13               -------------------------------------------------------------------------------- 0
AtBor4|Q9XI23               -------------------------------------------------------------------------------- 0
AtBor5|Q9SSG5               -------------------------------------------------------------------------------- 0
AtBor6|Q3E954               -------------------------------------------------------------------------------- 0
AtBor7|Q9SUU1               -------------------------------------------------------------------------------- 0
OsBor1|Q2QNH0               -------------------------------------------------------------------------------- 0
OsBor2|Q1ZYR7               -------------------------------------------------------------------------------- 0
OsBor3|Q7X9F3               -------------------------------------------------------------------------------- 0
OsBor4|Q1ZYR6               -------------------------------------------------------------------------------- 0
TaBor1.1|A0A060PT44         -------------------------------------------------------------------------------- 0
TaBor1.2|A0A060PVQ3         -------------------------------------------------------------------------------- 0
TaBor1.3|W5FNG8             -------------------------------------------------------------------------------- 0
ScBor1|P53838               -------------------------------------------------------------------------------- 0
SLC4A11|Q8NBS3              CVLLHTSRKYLKLKNFKEE--------------IRAHRDLDGFLAQASIVLNETATSLDNVL------------------ 136
SLC4A1/AE1|P02730           ------AARWVQLEENLGE-NGAWGRPHLSHLTFWSLLELRRVFTKGTVLLDLQETSLAGVANQLLDRFIFEDQIRPQDR 150
SLC4A2/AE2|P04920           ------TARWIKFEEDVEEETERWGKPHVASLSFRSLLELRRTLAHGAVLLDLDQQTLPGVAHQVVEQMVISDQIKAEDR 416
SLC4A3/AE3|P48751           ------TARWIKFEEDVEEETERWGKPHVASLSFRSLLELRRTIAHGAALLDLEQTTLPGIAHLVVETMIVSDQIRPEDR 414
SLC4A4/NBCe1|Q9Y6R1         ------TARWIKFEEKVEQGGERWSKPHVATLSLHSLFELRTCMEKGSIMLDREASSLPQLVEMIVDHQIETGLLKPELK 201
SLC4A5/NBCe2|Q9BY07         ------SARWIKFEEKVEEGGERWSKPHVSTLSLHSLFELRTCLQTGTVLLDLDSGSLPQIIDDVIEKQIEDGLLRPELR 205
SLC4A7/NBCn1|Q9Y6M7         ------TARWLKFEEDVEDGGDRWSKPYVATLSLHSLFELRSCILNGTVMLDMRASTLDEIADMVLDNMIASGQLDESIR 205
SLC4A8/NDCBE|Q2Y0W8         ------TARWLKFEEDVEDGGERWSKPYVATLSLHSLFELRSCLINGTVLLDMHANSIEEISDLILDQQELSSDLNDSMR 209
SLC4A9/AE4|Q96Q91           TLSTHLHHRWVLFEEKLEVAAGRWSAPHVPTLALPSLQKLRSLLAEGLVLLDCPAQSLLELVEQVTR----VESLSPELR 168
SLC4A10/NBCn2|Q6U841        ------TARWLKFEEDVEDGGERWSKPYVATLSLHSLFELRSCILNGTVLLDMHANTLEEIADMVLDQQVSSGQLNEDVR 210
```

```
AtBor1|Q8VYR7             -------------------------------------------------------------------------------- 0
AtBor2|Q9M1P7             -------------------------------------------------------------------------------- 0
AtBor3|Q93Z13             -------------------------------------------------------------------------------- 0
AtBor4|Q9XI23             -------------------------------------------------------------------------------- 0
AtBor5|Q9SSG5             -------------------------------------------------------------------------------- 0
AtBor6|Q3E954             -------------------------------------------------------------------------------- 0
AtBor7|Q9SUU1             -------------------------------------------------------------------------------- 0
OsBor1|Q2QNH0             -------------------------------------------------------------------------------- 0
OsBor2|Q1ZYR7             -------------------------------------------------------------------------------- 0
OsBor3|Q7X9F3             -------------------------------------------------------------------------------- 0
OsBor4|Q1ZYR6             -------------------------------------------------------------------------------- 0
TaBor1.1|A0A060PT44       -------------------------------------------------------------------------------- 0
TaBor1.2|A0A060PVQ3       -------------------------------------------------------------------------------- 0
TaBor1.3|W5FNG8           -------------------------------------------------------------------------------- 0
ScBor1|P53838             -------------------------------------------------------------------------------- 0
SLC4A11|Q8NBS3            ----RTMLRRFARDPDN------------------------------------NEPNCN--------------------- 155
SLC4A1/AE1|P02730         EELLRALLLKHSHAGELEALGGVKPAVLT---------RSGDP----------SQPLL---------------------- 189
SLC4A2/AE2|P04920         ANVLRALLLKHSHPSDEKDFS-FPRNISAGSLGSLLGHHHGQGAESDPHV---TEPLM---G-G---------------- 472
SLC4A3/AE3|P48751         ASVLRTLLLKHSHPNDDKDSGFFPRNPSSSSMNSVLGNHHPTPSHGPDGA---VPTM----ADD---------------- 471
SLC4A4/NBCe1|Q9Y6R1       DKVTYTLLRKHRHQTKK------SNLRSLADIGKTVSSASRMFTNPDNGSPAMTH-----R------------------- 251
SLC4A5/NBCe2|Q9BY07       ERVSYVLLRRHRHQTKK------PIHRSLADIGKSVSTTNRSPARSPGAGPSLHH-----STEDLRMRQSA--------- 265
SLC4A7/NBCn1|Q9Y6M7       ENVREALLKRHHHQNEKRFTSRIPLVRSFADIGKKHSDPHLLERNGEGLSASRHSLRTGLSASNLSLRGESPLSLLLGHL 285
SLC4A8/NDCBE|Q2Y0W8       VKVREALLKKHHHQNEKKRNNLIPIVRSFAEVGKKQSDPHLMDKH-GQTVSPQSVPTTNLEVKN---------------- 272
SLC4A9/AE4|Q96Q91         GQLQALLLQRPQHYNQTTGTR--PCW-------------------------GSTHPRK---------------------- 199
SLC4A10/NBCn2|Q6U841      HRVHEALMKQHHHQNQKKLTNRIPIVRSFADIGKKQSEPNSMDKNAGQVVSPQSAPAC-VENKN---------------- 273

AtBor1|Q8VYR7             -------------------------------------------------------------------------------- 0
AtBor2|Q9M1P7             -------------------------------------------------------------------------------- 0
AtBor3|Q93Z13             -------------------------------------------------------------------------------- 0
AtBor4|Q9XI23             -------------------------------------------------------------------------------- 0
AtBor5|Q9SSG5             -------------------------------------------------------------------------------- 0
AtBor6|Q3E954             -------------------------------------------------------------------------------- 0
AtBor7|Q9SUU1             -------------------------------------------------------------------------------- 0
OsBor1|Q2QNH0             -------------------------------------------------------------------------------- 0
OsBor2|Q1ZYR7             -------------------------------------------------------------------------------- 0
OsBor3|Q7X9F3             -------------------------------------------------------------------------------- 0
OsBor4|Q1ZYR6             -------------------------------------------------------------------------------- 0
TaBor1.1|A0A060PT44       -------------------------------------------------------------------------------- 0
TaBor1.2|A0A060PVQ3       -------------------------------------------------------------------------------- 0
TaBor1.3|W5FNG8           -------------------------------------------------------------------------------- 0
ScBor1|P53838             -------------------------------------------------------------------------------- 0
SLC4A11|Q8NBS3            -------------------------------------------------------------------------------- 155
SLC4A1/AE1|P02730         --------------------------------------PQHSSL------------------------------------ 195
SLC4A2/AE2|P04920         -------VPE----------T---------------RLEVERER------------------------------------ 484
SLC4A3/AE3|P48751         -----LGEPA----------P---------------LWPHDPDA------------------------------------ 485
SLC4A4/NBCe1|Q9Y6R1       -------------------------------------------------------------------------------- 251
SLC4A5/NBCe2|Q9BY07       -------------------------------------------------------------------------------- 265
SLC4A7/NBCn1|Q9Y6M7       LPSSRAGTPAGSRCTTPVPTPQNSPPSSPSISRLTSRSSQESQRQAPELLVSPASDDIPTVVIHPPEEDLEAALKGEEQK 365
SLC4A8/NDCBE|Q2Y0W8       ----------GVNCEH-------------------------------------------------SP------------- 280
SLC4A9/AE4|Q96Q91         -------------------------------------------------------------------------------- 199
SLC4A10/NBCn2|Q6U841      ----------DVSREN-------------------------------------------------STVDFSKGLGGQQKG 294

AtBor1|Q8VYR7             -------------------------------------------------------------------------------- 0
AtBor2|Q9M1P7             -------------------------------------------------------------------------------- 0
AtBor3|Q93Z13             -------------------------------------------------------------------------------- 0
AtBor4|Q9XI23             -------------------------------------------------------------------------------- 0
AtBor5|Q9SSG5             -------------------------------------------------------------------------------- 0
AtBor6|Q3E954             -------------------------------------------------------------------------------- 0
AtBor7|Q9SUU1             -------------------------------------------------------------------------------- 0
OsBor1|Q2QNH0             -------------------------------------------------------------------------------- 0
OsBor2|Q1ZYR7             -------------------------------------------------------------------------------- 0
OsBor3|Q7X9F3             -------------------------------------------------------------------------------- 0
OsBor4|Q1ZYR6             -------------------------------------------------------------------------------- 0
TaBor1.1|A0A060PT44       -------------------------------------------------------------------------------- 0
TaBor1.2|A0A060PVQ3       -------------------------------------------------------------------------------- 0
TaBor1.3|W5FNG8           -------------------------------------------------------------------------------- 0
ScBor1|P53838             -------------------------------------------------------------------------------- 0
SLC4A11|Q8NBS3            ----LDLLMAMLFTDAGAP--------------------------M--RGKVHLLSDTIQGVTATVTGVRYQQSWLCIIC 203
SLC4A1/AE1|P02730         -----E-------TQLFCEQ---------------------GDGGTEGHSPSGILEKIPPDSEATL----------VLVG 232
SLC4A2/AE2|P04920         -----E------LPPPAPPA---------------------GITRSKSKHELKLLEKIPENAEATV----------VLVG 522
SLC4A3/AE3|P48751         -----K------EKPLHMPG---------------------G--DGHRGKSLKLLEKIPEDAEATV----------VLVG 521
SLC4A4/NBCe1|Q9Y6R1       -------------------------------NLTSSSLNDISDKPEKDQLKNKFMKKLPRDAEASN----------VLVG 290
SLC4A5/NBCe2|Q9BY07       -------------------------NYGRLCHAQSRSMNDISLTPNTDQRKNKFMKKIPKDSEASN----------VLVG 310
SLC4A7/NBCn1|Q9Y6M7       NEENVDLTPGILASPQSAPGNLDNSKSGEIKGNGSGGSRE-NSTVDFSKVDMNFMRKIPTGAEASN----------VLVG 434
SLC4A8/NDCBE|Q2Y0W8       --------------------------------------------VDLSKVDLHFMKKIPTGAEASN----------VLVG 306
SLC4A9/AE4|Q96Q91         -------------------------------------ASDNEEAPLREQCQNPLRQKLPPGAEAGT----------VLAG 232
SLC4A10/NBCn2|Q6U841      H---------------TSP-----------CGMKQRHEKG-PPHQQEREVDLHFMKKIPPGAEASN----------ILVG 337
```

```
AtBor1|Q8VYR7                --------------------------------------------------------------------------------  0
AtBor2|Q9M1P7                --------------------------------------------------------------------------------  0
AtBor3|Q93Z13                --------------------------------------------------------------------------------  0
AtBor4|Q9XI23                --------------------------------------------------------------------------------  0
AtBor5|Q9SSG5                --------------------------------------------------------------------------------  0
AtBor6|Q3E954                --------------------------------------------------------------------------------  0
AtBor7|Q9SUU1                --------------------------------------------------------------------------------  0
OsBor1|Q2QNH0                --------------------------------------------------------------------------------  0
OsBor2|Q1ZYR7                --------------------------------------------------------------------------------  0
OsBor3|Q7X9F3                --------------------------------------------------------------------------------  0
OsBor4|Q1ZYR6                --------------------------------------------------------------------------------  0
TaBor1.1|A0A060PT44          --------------------------------------------------------------------------------  0
TaBor1.2|A0A060PVQ3          --------------------------------------------------------------------------------  0
TaBor1.3|W5FNG8              --------------------------------------------------------------------------------  0
ScBor1|P53838                --------------------------------------------------------------------------------  0
SLC4A11|Q8NBS3               TMKALQKRHVCISRL---VRPQNWGENSCEVRFVILVLAPPKMKSTKTAMEVARTFATMFSDIAFRQKLLETRTEEEFKE  280
SLC4A1/AE1|P02730            RADFLEQPVLGFVRLQEAAELEA-VELPVPIRFLFVLLGPEAP--HIDYTQLGRAAATLMSERVFRIDAYMAQSRGELLH  309
SLC4A2/AE2|P04920            CVEFLSRPTMAFVRLREAVELDAVLEVPVPVRFLFLLLGPSSA--NMDYHEIGRSISTLMSDKQFHEAAYLADEREDLLT  600
SLC4A3/AE3|P48751            CVPFLEQPAAAFVRLNEAVLLESVLEVPVPVRFLFVMLGPSHT--STDYHELGRSIATLMSDKLFHEAAYQADDRQDLLS  599
SLC4A4/NBCe1|Q9Y6R1          EVDFLDTPFIAFVRLQQAVMLGALTEVPVPTRFLFILLGPKGK--AKSYHEIGRAIATLMSDEVFHDIAYKAKDRHDLIA  368
SLC4A5/NBCe2|Q9BY07          EVDFLDQPFIAFVRLIQSAMLGGVTEVPVPTRFLFILLGPSGR--AKSYNEIGRAIATLMVDDLFSDVAYKARNREDLIA  388
SLC4A7/NBCn1|Q9Y6M7          EVDFLERPIIAFVRLAPAVLLTGLTEVPVPTRFLFLLLGPAGK--APQYHEIGRSIATLMTDEIFHDVAYKAKDRNDLLS  512
SLC4A8/NDCBE|Q2Y0W8          EVDILDRPIVAFVRLSPAVLLSGLTEVPIPTRFLFILLGPVGK--GQQYHEIGRSMATIMTDEIFHDVAYKAKERDDLLA  384
SLC4A9/AE4|Q96Q91            ELGFLAQPLGAFVRLRNPVVLGSLTEVSLPSRFFCLLLGPCML--GKGYHEMGRAAAVLLSDPQFQWSVRRASNLHDLLA  310
SLC4A10/NBCn2|Q6U841         ELEFLDRTVVAFVRLSPAVLLQGLAEVPIPTRFLFILLGPLGK--GQQYHEIGRSIATLMTDEVFHDVAYKAKDRNDLVS  415

AtBor1|Q8VYR7                --------------------------------------------------------------------------------  0
AtBor2|Q9M1P7                --------------------------------------------------------------------------------  0
AtBor3|Q93Z13                --------------------------------------------------------------------------------  0
AtBor4|Q9XI23                --------------------------------------------------------------------------------  0
AtBor5|Q9SSG5                --------------------------------------------------------------------------------  0
AtBor6|Q3E954                --------------------------------------------------------------------------------  0
AtBor7|Q9SUU1                --------------------------------------------------------------------------------  0
OsBor1|Q2QNH0                --------------------------------------------------------------------------------  0
OsBor2|Q1ZYR7                --------------------------------------------------------------------------------  0
OsBor3|Q7X9F3                --------------------------------------------------------------------------------  0
OsBor4|Q1ZYR6                --------------------------------------------------------------------------------  0
TaBor1.1|A0A060PT44          --------------------------------------------------------------------------------  0
TaBor1.2|A0A060PVQ3          --------------------------------------------------------------------------------  0
TaBor1.3|W5FNG8              --------------------------------------------------------------------------------  0
ScBor1|P53838                -----------------------------MSNESTRVTVSRG---CTASDECAQA-L----------------ERTNDEL  31
SLC4A11|Q8NBS3               ALVHQRQLLTMVSHGPVAPRTKERSTVSLPA-------------------------------------------------  311
SLC4A1/AE1|P02730            SLEGFLDCSLVLPPTDAPSEQALLSLVPVQRELLRR----RYQSSPAKPDSSFYKGL-----------------------  362
SLC4A2/AE2|P04920            AINAFLDCSVVLPPSEVQGEELLRSVAHFQRQMLKK----REEQGRLLP---TGAGLEPKS------------------A  655
SLC4A3/AE3|P48751            AISEFLDGSIVIPPSEVEGRDLLRSVAAFQRELLRK----RREREQTKVEMTTRGGYTA--------------------P  655
SLC4A4/NBCe1|Q9Y6R1          GIDEFLDEVIVLPPGEWDPAIRIEPPKSLPSSDKRKNMYSGGENVQMNGDTPHDG-GHGGGGH-----------------  430
SLC4A5/NBCe2|Q9BY07          GIDEFLDEVIVLPPGEWDPNIRIEPPKKVPSADKRKSVFSLAELGQMNGSVGGGG-GAPGGGNGGGGGGGSGGGAGSGGA  467
SLC4A7/NBCn1|Q9Y6M7          GIDEFLDQVTVLPPGEWDPSIRIEPPKSVPSQEKRKIPV------FHNGSTPTLG-ETPKEA------------------  567
SLC4A8/NDCBE|Q2Y0W8          GIDEFLDQVTVLPPGEWDPSIRIEPPKNVPSQEKRKMPG------VPNGNVCHIE-QEP--H------------------  437
SLC4A9/AE4|Q96Q91            ALDAFLEEVTVLPPGRWDPTARIPPPKCLPSQHKRLPSQQREIRG---PAVPRLT-------------------------  362
SLC4A10/NBCn2|Q6U841         GIDEFLDQVTVLPPGEWDPSIRIEPPKNVPSQEKRKIPA------VPNGTAAHGE-AEP--H------------------  468
```

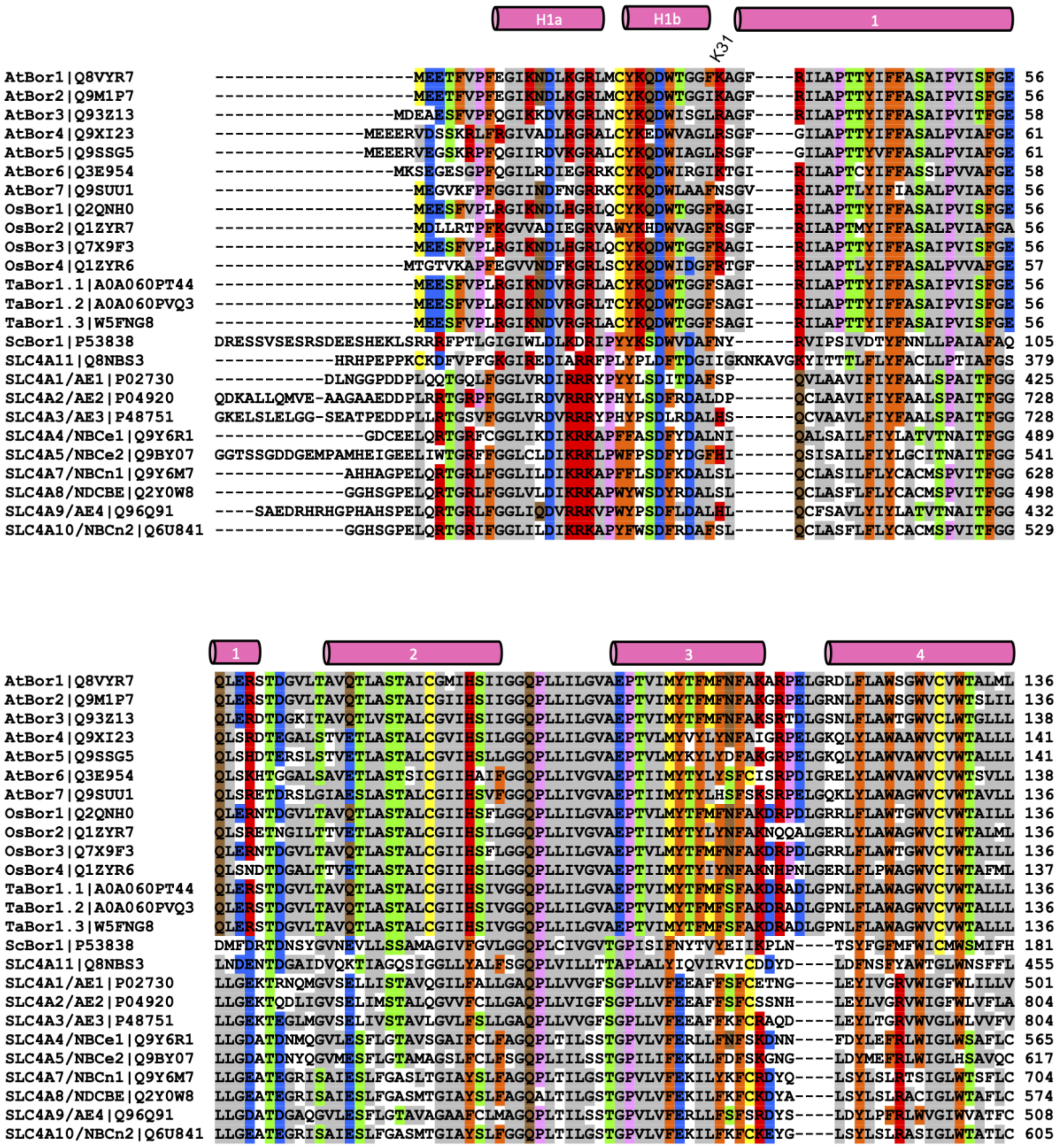
H1a
H1b
1
K31
AtBor1|Q8VYR7 --------------------MEETFVPFEGIKNDLKGRLMCYKQDWTGGFKAGF----RILAPTTYIFFASAIPVISFGE 56
AtBor2|Q9M1P7 --------------------MEETFVPFEGIKNDLKGRLMCYKQDWTGGIKAGF----RILAPTTYIFFASAIPVISFGE 56
AtBor3|Q93Z13 ------------------MDEAESFVPFQGIKKDVKGRLNCYKQDWISGLRAGF----RILAPTTYIFFASAIPVITFGE 58
AtBor4|Q9XI23 ---------------MEEERVDSSKRLFRGIVADLRGRALCYKEDWVAGLRSGF----GILAPTTYIFFASALPVIAFGE 61
AtBor5|Q9SSG5 ---------------MEEERVEGSKRPFQGIIRDVKGRALCYKQDWIAGLRSGF----GILAPTTYVFFASALPVIAFGE 61
AtBor6|Q3E954 ------------------MKSEGESGPFQGILRDIEGRRKCYKQDWIRGIKTGI----RILAPTCYIFFASSLPVVAFGE 58
AtBor7|Q9SUU1 --------------------MEGVKFPFGGIINDFNGRRKCYKQDWLAAFNSGV----RILAPTLYIFIASALPVIAFGE 56
OsBor1|Q2QNH0 --------------------MEESFVPLRGIKNDLHGRLQCYKQDWTGGFRAGI----RILAPTTYIFFASAIPVISFGE 56
OsBor2|Q1ZYR7 --------------------MDLLRTPFKGVVADIEGRVAWYKHDWVAGFRSGF----RILAPTMYIFFASALPVIAFGA 56
OsBor3|Q7X9F3 --------------------MEESFVPLRGIKNDLHGRLQCYKQDWTGGFRAGI----RILAPTTYIFFASAIPVISFGE 56
OsBor4|Q1ZYR6 -------------------MTGTVKAPFEGVVNDFKGRLSCYKQDWIDGFRTGF----RILAPTLYIFFASALPVVAFGE 57
TaBor1.1|A0A060PT44 --------------------MEESFVPLRGIKNDVRGRLACYKQDWTGGFSAGI----RILAPTTYIFFASAIPVISFGE 56
TaBor1.2|A0A060PVQ3 --------------------MEESFVPLRGIKNDVRGRLTCYKQDWTGGFSAGI----RILAPTTYIFFASAIPVISFGE 56
TaBor1.3|W5FNG8 --------------------MEESFVPLRGIKNDVRGRLACYKQDWTGGFSAGI----RILAPTTYIFFASAIPVISFGE 56
ScBor1|P53838 DRESSVSESRSDEESHEKLSRRRFPTLGIGIWLDLKDRIPYYKSDWVDAFNY------RVIPSIVDTYFNNLLPAIAFAQ 105
SLC4A11|Q8NBS3 ------------HRHPEPPKCKDFVPFGKGIREDIARRFPLYPLDFTDGIIGKNKAVGKYITTTLFLYFACLLPTIAFGS 379
SLC4A1/AE1|P02730 -----------DLNGGPDDPLQQTGQLFGGLVRDIRRRYPYYLSDITDAFSP------QVLAAVIFIYFAALSPAITFGG 425
SLC4A2/AE2|P04920 QDKALLQMVE-AAGAAEDDPLRRTGRPFGGLIRDVRRRYPHYLSDFRDALDP------QCLAAVIFIYFAALSPAITFGG 728
SLC4A3/AE3|P48751 GKELSLELGG-SEATPEDDPLLRTGSVFGGLVRDVRRRYPHYPSDLRDALHS------QCVAAVLFIYFAALSPAITFGG 728
SLC4A4/NBCe1|Q9Y6R1 ---------------GDCEELQRTGRFCGGLIKDIKRKAPFFASDFYDALNI------QALSAILFIYLATVTNAITFGG 489
SLC4A5/NBCe2|Q9BY07 GGTSSGDDGEMPAMHEIGEELIWTGRFFGGLCLDIKRKLPWFPSDFYDGFHI------QSISAILFIYLGCITNAITFGG 541
SLC4A7/NBCn1|Q9Y6M7 -------------AHHAGPELQRTGRLFGGLILDIKRKAPFFLSDFKDALSL------QCLASILFLYCACMSPVITFGG 628
SLC4A8/NDCBE|Q2Y0W8 -------------GGHSGPELQRTGRLFGGLVLDIKRKAPWYWSDYRDALSL------QCLASFLFLYCACMSPVITFGG 498
SLC4A9/AE4|Q96Q91 ----SAEDRHRHGPHAHSPELQRTGRLFGGLIQDVRRKVPWYPSDFLDALHL------QCFSAVLYIYLATVTNAITFGG 432
SLC4A10/NBCn2|Q6U841 -------------GGHSGPELQRTGRIFGGLILDIKRKAPYFWSDFRDAFSL------QCLASFLFLYCACMSPVITFGG 529
1
2
3
4
AtBor1|Q8VYR7 QLERSTDGVLTAVQTLASTAICGMIHSIIGGQPLLILGVAEPTVIMYTFMFNFAKARPELGRDLFLAWSGWVCVWTALML 136
AtBor2|Q9M1P7 QLERSTDGVLTAVQTLASTAICGIIHSIIGGQPLLILGVAEPTVIMYTFMFNFAKGRPELGRNLFLAWSGWVCVWTSLIL 136
AtBor3|Q93Z13 QLERDTDGKITAVQTLVSTALCGVIHSIIGGQPLLILGVAEPTVIMYTFMFNFAKSRTDLGSNLFLAWTGWVCLWTGLLL 138
AtBor4|Q9XI23 QLSRDTEGALSTVETLASTALCGVIHSILGGQPLLILGVAEPTVLMYVYLYNFAIGRPELGKQLYLAWAAWVCVWTALLL 141
AtBor5|Q9SSG5 QLSHDTERSLSTVETLASTALCGVIHSLLGGQPLLILGVAEPTVLMYKYLYDFAKGRPELGKQLYLAWVAWVCVWTALLL 141
AtBor6|Q3E954 QLSKHTGGALSAVETLASTSICGIIHAIFGGQPLLIVGVAEPTIIMYTYLYSFCISRPDIGRELYLAWVAWVCVWTSVLL 138
AtBor7|Q9SUU1 QLSRETDRSLGIAESLASTALCGIIHSVFGGQPLLIVGVAEPTIIMYTYLHSFSKSRPELGQKLYLAWAGWVCVWTAVLL 136
OsBor1|Q2QNH0 QLERNTDGVLTAVQTLASTALCGIIHSFLGGQPLLILGVAEPTVLMYTFMFNFAKDRPDLGRRLFLAWTGWVCVWTAILL 136
OsBor2|Q1ZYR7 QLSRETNGILTTVETLASTALCGIIHSILGGQPLLIVGVAEPTIIMYTYLYNFAKNQQALGERLYLAWAGWVCIWTALML 136
OsBor3|Q7X9F3 QLERNTDGVLTAVQTLASTALCGIIHSFLGGQPLLILGVAEPTVLMYTFMFNFAKDRPDLGRRLFLAWTGWVCVWTAILL 136
OsBor4|Q1ZYR6 QLSNDTDGALTTVETLASTAICGIIHSILGGQPLLIVGVAEPTIIMYTYIYNFAKNHPNLGERLFLPWAGWVCIWTAFML 137
TaBor1.1|A0A060PT44 QLERSTDGVLTAVQTLASTALCGIIHSIVGGQPLLILGVAEPTVIMYTFMFSFAKDRADLGPNLFLAWAGWVCVWTALLL 136
TaBor1.2|A0A060PVQ3 QLERSTDGVLTAVQTLASTALCGIIHSIVGGQPLLILGVAEPTVIMYTFMFSFAKDRADLGPNLFLAWAGWVCVWTALLL 136
TaBor1.3|W5FNG8 QLERSTDGVLTAVQTLASTALCGIIHSIVGGQPLLILGVAEPTVIMYTFMFSFAKDRADLGPNLFLAWAGWVCVWTALLL 136
ScBor1|P53838 DMFDRTDNSYGVNEVLLSSAMAGIVFGVLGGQPLCIVGVTGPISIFNYTVYEIIKPLN----TSYFGFMFWICMWSMIFH 181
SLC4A11|Q8NBS3 LNDENTDGAIDVQKTIAGQSIGGLLYALFSGQPLVILLTTAPLALYIQVIRVICDDYD----LDFNSFYAWTGLWNSFFL 455
SLC4A1/AE1|P02730 LLGEKTRNQMGVSELLISTAVQGILFALLGAQPLLVVGFSGPLLVFEEAFFSFCETNG----LEYIVGRVWIGFWLILLV 501
SLC4A2/AE2|P04920 LLGEKTQDLIGVSELIMSTALQGVVFCLLGAQPLLVIGFSGPLLVFEEAFFSFCSSNH----LEYLVGRVWIGFWLVFLA 804
SLC4A3/AE3|P48751 LLGEKTEGLMGVSELIVSTAVLGVLFSLLGAQPLLVVGFSGPLLVFEEAFFKFCRAQD----LEYLTGRVWVGLWLVVFV 804
SLC4A4/NBCe1|Q9Y6R1 LLGDATDNMQGVLESFLGTAVSGAIFCLFAGQPLTILSSTGPVLVFERLLFNFSKDNN----FDYLEFRLWIGLWSAFLC 565
SLC4A5/NBCe2|Q9BY07 LLGDATDNYQGVMESFLGTAMAGSLFCLFSGQPLIILSSTGPILIFEKLLFDFSKGNG----LDYMEFRLWIGLHSAVQC 617
SLC4A7/NBCn1|Q9Y6M7 LLGEATEGRISAIESLFGASLTGIAYSLFAGQPLTILGSTGPVLVFEKILYKFCRDYQ----LSYLSLRTSIGLWTSFLC 704
SLC4A8/NDCBE|Q2Y0W8 LLGEATEGRISAIESLFGASMTGIAYSLFAGQALTILGSTGPVLVFEKILFKFCKDYA----LSYLSLRACIGLWTAFLC 574
SLC4A9/AE4|Q96Q91 LLGDATDGAQGVLESFLGTAVAGAAFCLMAGQPLTILSSTGPVLVFERLLFSFSRDYS----LDYLPFRLWVGIWVATFC 508
SLC4A10/NBCn2|Q6U841 LLGEATEGRISAIESLFGASMTGIAYSLFGGQPLTILGSTGPVLVFEKILFKFCKEYG----LSYLSLRASIGLWTATLC 605

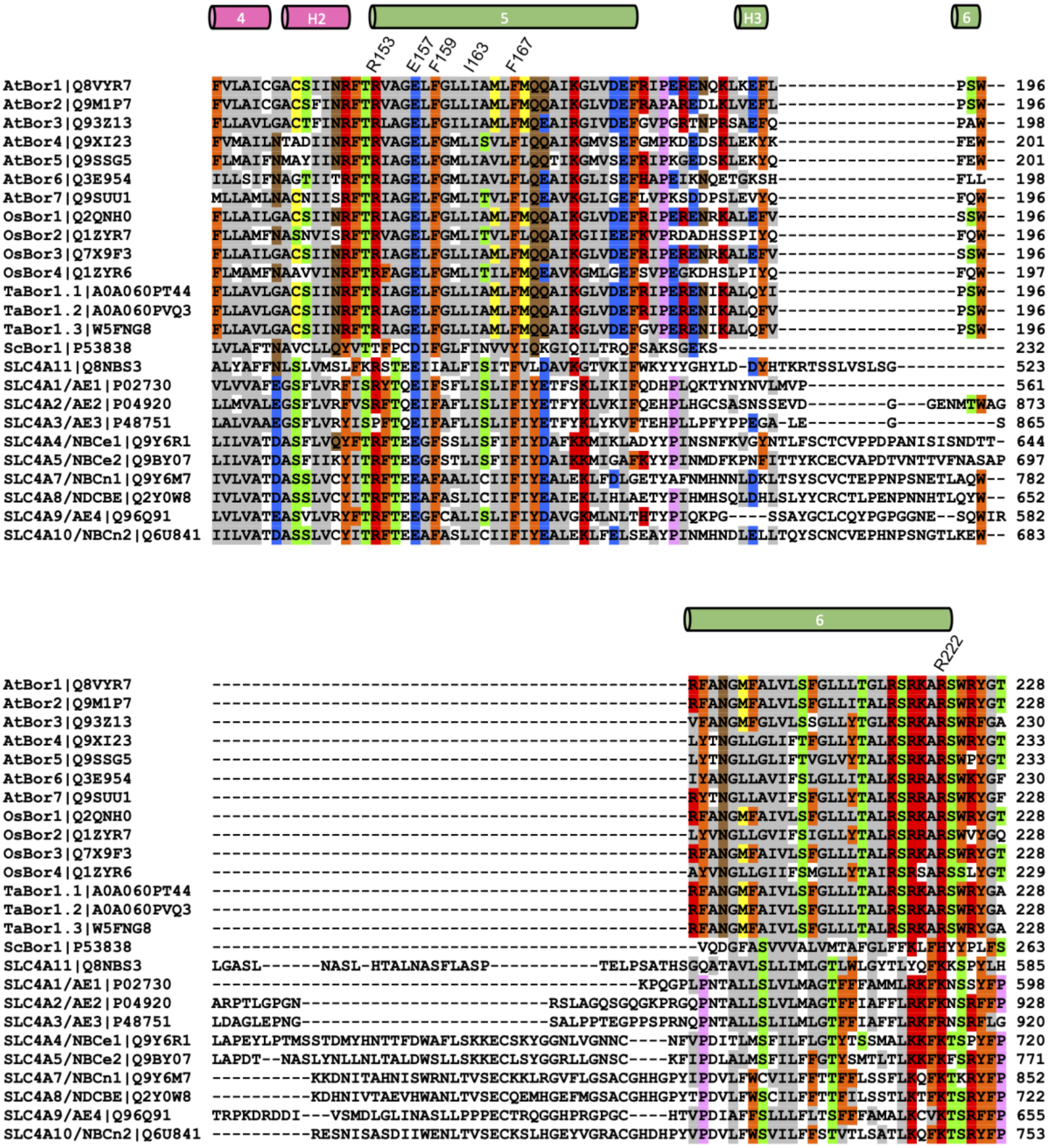
4
H2
5
H3
6
R153
E157
F159
I163
F167
AtBor1|Q8VYR7 196
AtBor2|Q9M1P7 196
AtBor3|Q93Z13 198
AtBor4|Q9XI23 201
AtBor5|Q9SSG5 201
AtBor6|Q3E954 198
AtBor7|Q9SUU1 196
OsBor1|Q2QNH0 196
OsBor2|Q1ZYR7 196
OsBor3|Q7X9F3 196
OsBor4|Q1ZYR6 197
TaBor1.1|A0A060PT44 196
TaBor1.2|A0A060PVQ3 196
TaBor1.3|W5FNG8 196
ScBor1|P53838 232
SLC4A11|Q8NBS3 523
SLC4A1/AE1|P02730 561
SLC4A2/AE2|P04920 873
SLC4A3/AE3|P48751 865
SLC4A4/NBCe1|Q9Y6R1 644
SLC4A5/NBCe2|Q9BY07 697
SLC4A7/NBCn1|Q9Y6M7 782
SLC4A8/NDCBE|Q2Y0W8 652
SLC4A9/AE4|Q96Q91 582
SLC4A10/NBCn2|Q6U841 683
6
R222
AtBor1|Q8VYR7 228
AtBor2|Q9M1P7 228
AtBor3|Q93Z13 230
AtBor4|Q9XI23 233
AtBor5|Q9SSG5 233
AtBor6|Q3E954 230
AtBor7|Q9SUU1 228
OsBor1|Q2QNH0 228
OsBor2|Q1ZYR7 228
OsBor3|Q7X9F3 228
OsBor4|Q1ZYR6 229
TaBor1.1|A0A060PT44 228
TaBor1.2|A0A060PVQ3 228
TaBor1.3|W5FNG8 228
ScBor1|P53838 263
SLC4A11|Q8NBS3 585
SLC4A1/AE1|P02730 598
SLC4A2/AE2|P04920 928
SLC4A3/AE3|P48751 920
SLC4A4/NBCe1|Q9Y6R1 720
SLC4A5/NBCe2|Q9BY07 771
SLC4A7/NBCn1|Q9Y6M7 852
SLC4A8/NDCBE|Q2Y0W8 722
SLC4A9/AE4|Q96Q91 655
SLC4A10/NBCn2|Q6U841 753

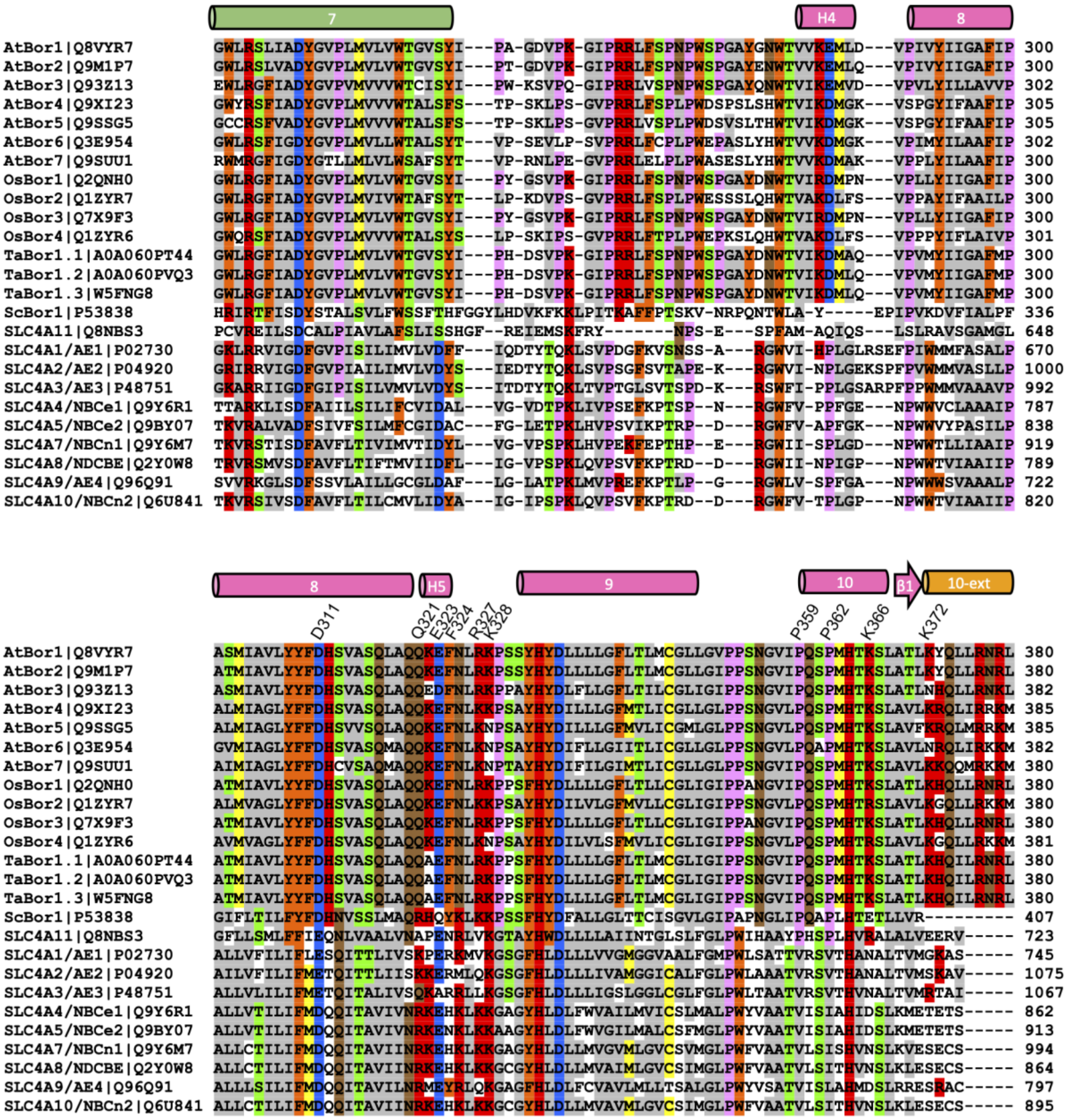

7
H4
8
AtBor1|Q8VYR7 GWLRSLIADYGVPLMVLVWTGVSYI---PA-GDVPK-GIPRRLFSPNPWSPGAYGNWTVVKEMLD---VPIVYIIGAFIP 300
AtBor2|Q9M1P7 GWLRSLVADYGVPLMVLVWTGVSYI---PT-GDVPK-GIPRRLFSPNPWSPGAYENWTVVKEMLQ---VPIVYIIGAFIP 300
AtBor3|Q93Z13 EWLRGFIADYGVPVMVVVWTCISYI---PW-KSVPQ-GIPRRLVSPNPWSPGAYQNWTVIKEMVD---VPVLYILLAVVP 302
AtBor4|Q9XI23 GWYRSFIADYGVPLMVVVWTALSFS---TP-SKLPS-GVPRRLFSPLPWDSPSLSHWTVIKDMGK---VSPGYIFAAFIP 305
AtBor5|Q9SSG5 GCCRSFVADYGVPLMVVVWTALSFS---TP-SKLPS-GVPRRLVSPLPWDSVSLTHWTVIKDMGK---VSPGYIFAAFIP 305
AtBor6|Q3E954 GWLRSFIGDYGVPLMVLLWTALSYT---VP-SEVLP-SVPRRLFCPLPWEPASLYHWTVVKDMGK---VPIMYILAAFIP 302
AtBor7|Q9SUU1 RWMRGFIGDYGTLLMLVLWSAFSYT---VP-RNLPE-GVPRRLELPLPWASESLYHWTVVKDMAK---VPPLYILAAFIP 300
OsBor1|Q2QNH0 GWLRGFIADYGVPLMVLVWTGVSYI---PY-GSVPK-GIPRRLFSPNPWSPGAYDNWTVIRDMPN---VPLLYIIGAFIP 300
OsBor2|Q1ZYR7 GWLRGFIADYGVPLMVIVWTAFSYT---LP-KDVPS-GVPRRLFSPLPWESSSLQHWTVAKDLFS---VPPAYIFAAILP 300
OsBor3|Q7X9F3 GWLRGFIADYGVPLMVLVWTGVSYI---PY-GSVPK-GIPRRLFSPNPWSPGAYDNWTVIRDMPN---VPLLYIIGAFIP 300
OsBor4|Q1ZYR6 GWQRSFIADYGVPLMVVVWTALSYS---LP-SKIPS-GVPRRLFTPLPWEPKSLQHWTVAKDLFS---VPPPYIFLAIVP 301
TaBor1.1|A0A060PT44 GWLRGFIADYGVPLMVLVWTGVSYI---PH-DSVPK-GIPRRLFSPNPWSPGAYDNWTVIKDMAQ---VPVMYIIGAFMP 300
TaBor1.2|A0A060PVQ3 GWLRGFIADYGVPLMVLVWTGVSYI---PH-DSVPK-GIPRRLFSPNPWSPGAYDNWTVIKDMLQ---VPVMYIIGAFMP 300
TaBor1.3|W5FNG8 GWLRGFIADYGVPLMVLVWTGVSYI---PH-DSVPK-GIPRRLFSPNPWSPGAYDNWTVIKDMLQ---VPVMYIIGAFMP 300
ScBor1|P53838 HRIRTFISDYSTALSVLFWSSFTHFGGYLHDVKFKKLPITKAFFPTSKV-NRPQNTWLA-Y-----EPIPVKDVFIALPF 336
SLC4A11|Q8NBS3 PCVREILSDCALPIAVLAFSLISSHGF--REIEMSKFRY-------NPS-E---SPFAM-AQIQS---LSLRAVSGAMGL 648
SLC4A1/AE1|P02730 GKLRRVIGDFGVPISILIMVLVDFF---IQDTYTQKLSVPDGFKVSNSS-A---RGWVI-HPLGLRSEFPIWMMFASALP 670
SLC4A2/AE2|P04920 GRIRRVIGDFGVPIAILIMVLVDYS---IEDTYTQKLSVPSGFSVTAPE-K---RGWVI-NPLGEKSPFPVWMMVASLLP 1000
SLC4A3/AE3|P48751 GKARRIIGDFGIPISILVMVLVDYS---ITDTYTQKLTVPTGLSVTSPD-K---RSWFI-PPLGSARPFPPWMMVAAAVP 992
SLC4A4/NBCe1|Q9Y6R1 TTARKLISDFAIILSILIFCVIDAL---VG-VDTPKLIVPSEFKPTSP--N---RGWFV-PPFGE---NPWWVCLAAAIP 787
SLC4A5/NBCe2|Q9BY07 TKVRALVADFSIVFSILMFCGIDAC---FG-LETPKLHVPSVIKPTRP--D---RGWFV-APFGK---NPWWVYPASILP 838
SLC4A7/NBCn1|Q9Y6M7 TKVRSTISDFAVFLTIVIMVTIDYL---VG-VPSPKLHVPEKFEPTHP--E---RGWII-SPLGD---NPWWTLLIAAIP 919
SLC4A8/NDCBE|Q2Y0W8 TRVRSMVSDFAVFLTIFTMVIIDFL---IG-VPSPKLQVPSVFKPTRD--D---RGWII-NPIGP---NPWWTVIAAIIP 789
SLC4A9/AE4|Q96Q91 SVVRKGLSDFSSVLAILLGCGLDAF---LG-LATPKLMVPREFKPTLP--G---RGWLV-SPFGA---NPWWWSVAAALP 722
SLC4A10/NBCn2|Q6U841 TKVRSIVSDFAVFLTILCMVLIDYA---IG-IPSPKLQVPSVFKPTRD--D---RGWFV-TPLGP---NPWWTVIAAIIP 820
8
H5
9
10
β1
10-ext
D311
Q321
E323
F324
R327
K328
P359
P362
K366
K372
AtBor1|Q8VYR7 ASMIAVLYYFDHSVASQLAQQKEFNLRKPSSYHYDLLLLGFLTLMCGLLGVPPSNGVIPQSPMHTKSLATLKYQLLRNRL 380
AtBor2|Q9M1P7 ATMIAVLYYFDHSVASQLAQQKEFNLRKPSSYHYDLLLLGFLTLMCGLLGIPPSNGVIPQSPMHTKSLATLKYQLLRNRL 380
AtBor3|Q93Z13 ASMIAVLYYFDHSVASQLAQQEDFNLRKPPAYHYDLFLLGFLTILCGLIGIPPSNGVIPQSPMHTKSLATLNHQLLRNKL 382
AtBor4|Q9XI23 ALMIAGLYFFDHSVASQLAQQKEFNLKKPSAYHYDILLLGFMTLICGLLGLPPSNGVLPQSPMHTKSLAVLKRQLIRRKM 385
AtBor5|Q9SSG5 ALMIAGLYFFDHSVVSQLAQQKEFNLKNPSAYHYDILLLGFMVLICGMLGLPPSNGVLPQSPMHTKSLAVFKRQLMRRKM 385
AtBor6|Q3E954 GVMIAGLYFFDHSVASQMAQQKEFNLKNPSAYHYDIFLLGIITLICGLLGLPPSNGVLPQAPMHTKSLAVLNRQLIRKKM 382
AtBor7|Q9SUU1 AIMIAGLYFFDHCVSAQMAQQKEFNLKNPTAYHYDIFILGIMTLICGLLGLPPSNGVIPQSPMHTKSLAVLKKQQMRKKM 380
OsBor1|Q2QNH0 ATMIAVLYYFDHSVASQLAQQKEFNLRKPPSFHYDLLLLGFLTLLCGLIGIPPANGVIPQSPMHTKSLATLKHQLLRNRL 380
OsBor2|Q1ZYR7 ALMVAGLYFFDHSVASQLAQQKEFNLKKPSAYHYDILVLGFMVLLCGLIGIPPSNGVLPQSPMHTRSLAVLKGQLLRKKM 380
OsBor3|Q7X9F3 ATMIAVLYYFDHSVASQLAQQKEFNLRKPPSFHYDLLLLGFLTLLCGLIGIPPANGVIPQSPMHTKSLATLKHQLLRNRL 380
OsBor4|Q1ZYR6 AVMVAGLYFFDHSVASQLAQQKEFNLKNPSAYHYDILVLSFMVLICGLIGIPPSNGVLPQSPMHTRSLAVLKGQLLRKKM 381
TaBor1.1|A0A060PT44 ATMIAVLYYFDHSVASQLAQQAEFNLRKPPSFHYDLLLLGFLTLMCGLIGIPPSNGVIPQSPMHTKSLATLKHQILRNRL 380
TaBor1.2|A0A060PVQ3 ATMIAVLYYFDHSVASQLAQQAEFNLRKPPSFHYDLLLLGFLTLMCGLIGIPPSNGVIPQSPMHTKSLATLKHQILRNRL 380
TaBor1.3|W5FNG8 ATMIAVLYYFDHSVASQLAQQAEFNLRKPPSFHYDLLLLGFLTLMCGLIGIPPSNGVIPQSPMHTKSLATLKHQILRNRL 380
ScBor1|P53838 GIFLTILFYFDHNVSSLMAQRHQYKLKKPSSFHYDFALLGLTTCISGVLGIPAPNGLIPQAPLHTETLLVR--------- 407
SLC4A11|Q8NBS3 GFLLSMLFFIEQNLVAALVNAPENRLVKGTAYHWDLLLLAIINTGLSLFGLPWIHAAYPHSPLHVRALALVEERV----- 723
SLC4A1/AE1|P02730 ALLVFILIFLESQITTLIVSKPERKMVKGSGFHLDLLLVVGMGGVAALFGMPWLSATTVRSVTHANALTVMGKAS----- 745
SLC4A2/AE2|P04920 AILVFILIFMETQITTLIISKKERMLQKGSGFHLDLLLIVAMGGICALFGLPWLAAATVRSVTHANALTVMSKAV----- 1075
SLC4A3/AE3|P48751 ALLVLILIFMETQITALIVSQKARRLLKGSGFHLDLLLIGSLGGLCGLFGLPWLTAATVRSVTHVNALTVMRTAI----- 1067
SLC4A4/NBCe1|Q9Y6R1 ALLVTILIFMDQQITAVIVNRKEHKLKKGAGYHLDLFWVAILMVICSLMALPWYVAATVISIAHIDSLKMETETS----- 862
SLC4A5/NBCe2|Q9BY07 ALLVTILIFMDQQITAVIVNRKENKLKKAAGYHLDLFWVGILMALCSFMGLPWYVAATVISIAHIDSLKMETETS----- 913
SLC4A7/NBCn1|Q9Y6M7 ALLCTILIFMDQQITAVIINRKEHKLKKGAGYHLDLLMVGVMLGVCSVMGLPWFVAATVLSISHVNSLKVESECS----- 994
SLC4A8/NDCBE|Q2Y0W8 ALLCTILIFMDQQITAVIINRKEHKLKKGCGYHLDLLMVAIMLGVCSIMGLPWFVAATVLSITHVNSLKLESECS----- 864
SLC4A9/AE4|Q96Q91 ALLLSILIFMDQQITAVILNRMEYRLQKGAGFHLDLFCVAVLMLLTSALGLPWYVSATVISLAHMDSLRRESRAC----- 797
SLC4A10/NBCn2|Q6U841 ALLCTILIFMDQQITAVIINRKEHKLKKGCGYHLDLLMVAVMLGVCSIMGLPWFVAATVLSITHVNSLKLESECS----- 895

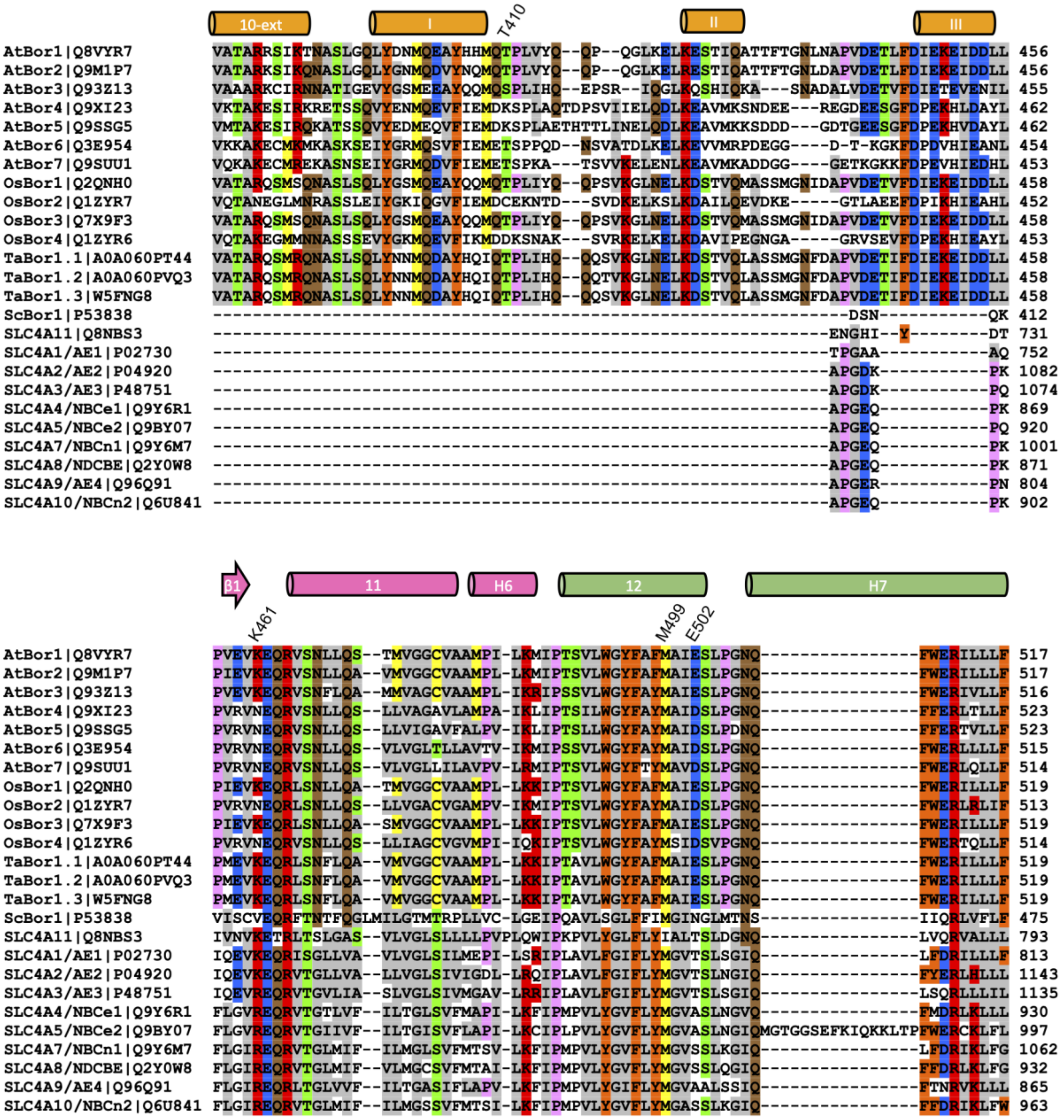

10-ext
I
T410
II
III
AtBor1|Q8VYR7 VATARRSIKTNASLGQLYDNMQEAYHHMQTPLVYQ--QP--QGLKELKESTIQATTFTGNLNAPVDETLFDIEKEIDDLL 456
AtBor2|Q9M1P7 VATARKSIKQNASLGQLYGNMQDVYNQMQTPLVYQ--QP--QGLKELRESTIQATTFTGNLDAPVDETLFDIEKEIDDLL 456
AtBor3|Q93Z13 VAAARKCIRNNATIGEVYGSMEEAYQQMQSPLIHQ--EPSR--IQGLKQSHIQKA---SNADALVDETVFDIETEVENIL 455
AtBor4|Q9XI23 VKTAKESIRKRETSSQVYENMQEVFIEMDKSPLAQTDPSVIIELQDLKEAVMKSNDEE---REGDEESGFDPEKHLDAYL 462
AtBor5|Q9SSG5 VMTAKESIRQKATSSQVYEDMEQVFIEMDKSPLAETHTTLINELQDLKEAVMKKSDDD---GDTGEESGFDPEKHVDAYL 462
AtBor6|Q3E954 VKKAKECMKMKASKSEIYGRMQSVFIEMETSPPQD--NSVATDLKELKEVVMRPDEGG----D-T-KGKFDPDVHIEANL 454
AtBor7|Q9SUU1 VQKAKECMREKASNSEIYGRMQDVFIEMETSPKA---TSVVKELENLKEAVMKADDGG----GETKGKKFDPEVHIEDHL 453
OsBor1|Q2QNH0 VATARQSMSQNASLSQLYGSMQEAYQQMQTPLIYQ--QPSVKGLNELKDSTVQMASSMGNIDAPVDETVFDIEKEIDDLL 458
OsBor2|Q1ZYR7 VQTANEGLMNRASSLEIYGKIQGVFIEMDCEKNTD---SVDKELKSLKDAILQEVDKE-----GTLAEEFDPIKHIEAHL 452
OsBor3|Q7X9F3 VATARQSMSQNASLSQLYGSMQEAYQQMQTPLIYQ--QPSVKGLNELKDSTVQMASSMGNIDAPVDETVFDIEKEIDDLL 458
OsBor4|Q1ZYR6 VQTAKEGMMNNASSSEVYGKMQEVFIKMDDKSNAK---SVRKELKELKDAVIPEGNGA-----GRVSEVFDPEKHIEAYL 453
TaBor1.1|A0A060PT44 VATARQSMRQNASLSQLYNNMQDAYHQIQTPLIHQ--QQSVKGLNELKDSTVQLASSMGNFDAPVDETIFDIEKEIDDLL 458
TaBor1.2|A0A060PVQ3 VATARQSMRQNASLSQLYNNMQDAYHQIQTPLIHQ--QQTVKGLNELKDSTVQLASSMGNFDAPVDETIFDIEKEIDDLL 458
TaBor1.3|W5FNG8 VATARQSMRQNASLSQLYNNMQDAYHQIQTPLIHQ--QQSVKGLNELKDSTVQLASSMGNFDAPVDETIFDIEKEIDDLL 458
ScBor1|P53838 ---DSN------------QK 412
SLC4A11|Q8NBS3 ---ENGHI--Y---------DT 731
SLC4A1/AE1|P02730 ---TPGAA------------AQ 752
SLC4A2/AE2|P04920 ---APGDK------------PK 1082
SLC4A3/AE3|P48751 ---APGDK------------PQ 1074
SLC4A4/NBCe1|Q9Y6R1 ---APGEQ------------PK 869
SLC4A5/NBCe2|Q9BY07 ---APGEQ------------PQ 920
SLC4A7/NBCn1|Q9Y6M7 ---APGEQ------------PK 1001
SLC4A8/NDCBE|Q2Y0W8 ---APGEQ------------PK 871
SLC4A9/AE4|Q96Q91 ---APGER------------PN 804
SLC4A10/NBCn2|Q6U841 ---APGEQ------------PK 902
β1
11
H6
12
H7
K461
M499
E502
AtBor1|Q8VYR7 PVEVKEQRVSNLLQS--TMVGGCVAAMPI-LKMIPTSVLWGYFAFMAIESLPGNQ----------------FWERILLLF 517
AtBor2|Q9M1P7 PIEVKEQRVSNLLQA--VMVGGCVAAMPL-LKMIPTSVLWGYFAFMAIESLPGNQ----------------FWERILLLF 517
AtBor3|Q93Z13 PVEVKEQRVSNFLQA--MMVAGCVAAMPL-IKRIPSSVLWGYFAYMAIESLPGNQ----------------FWERIVLLF 516
AtBor4|Q9XI23 PVRVNEQRVSNLLQS--LLVAGAVLAMPA-IKLIPTSILWGYFAYMAIDSLPGNQ----------------FFERLTLLF 523
AtBor5|Q9SSG5 PVRVNEQRVSNLLQS--LLVIGAVFALPV-IKLIPTSLLWGYFAYMAIDSLPDNQ----------------FFERTVLLF 523
AtBor6|Q3E954 PVRVNEQRVSNLLQS--VLVGLTLLAVTV-IKMIPSSVLWGYFAYMAIDSLPGNQ----------------FWERLLLLF 515
AtBor7|Q9SUU1 PVRVNEQRVSNLLQS--VLVGLLILAVPV-LRMIPTSVLWGYFTYMAVDSLPGNQ----------------FWERLQLLF 514
OsBor1|Q2QNH0 PIEVKEQRLSNLLQA--TMVGGCVAAMPL-LKKIPTSVLWGYFAFMAIESLPGNQ----------------FWERILLLF 519
OsBor2|Q1ZYR7 PVRVNEQRLSNLLQS--LLVGACVGAMPV-IKMIPTSVLWGYFAYMAIDSLPGNQ----------------FWERLRLIF 513
OsBor3|Q7X9F3 PIEVKEQRLSNLLQA--SMVGGCVAAMPL-LKKIPTSVLWGYFAFMAIESLPGNQ----------------FWERILLLF 519
OsBor4|Q1ZYR6 PVRVNEQRLSNLLQS--LLIAGCVGVMPI-IQKIPTSVLWGYFAYMSIDSVPGNQ----------------FWERTQLLF 514
TaBor1.1|A0A060PT44 PMEVKEQRLSNFLQA--VMVGGCVAAMPL-LKKIPTAVLWGYFAFMAIESLPGNQ----------------FWERILLLF 519
TaBor1.2|A0A060PVQ3 PMEVKEQRLSNFLQA--VMVGGCVAAMPL-LKKIPTAVLWGYFAFMAIESLPGNQ----------------FWERILLLF 519
TaBor1.3|W5FNG8 PMEVKEQRLSNFLQA--VMVGGCVAAMPL-LKKIPTAVLWGYFAFMAIESLPGNQ----------------FWERILLLF 519
ScBor1|P53838 VISCVEQRFTNTFQGLMILGTMTRPLLVC-LGEIPQAVLSGLFFIMGINGLMTNS----------------IIQRLVFLF 475
SLC4A11|Q8NBS3 IVNVKETRLTSLGAS--VLVGLSLLLLPVPLQWIPKPVLYGLFLYIALTSLDGNQ----------------LVQRVALLL 793
SLC4A1/AE1|P02730 IQEVKEQRISGLLVA--VLVGLSILMEPI-LSRIPLAVLFGIFLYMGVTSLSGIQ----------------LFDRILLLF 813
SLC4A2/AE2|P04920 IQEVKEQRVTGLLVA--LLVGLSIVIGDL-LRQIPLAVLFGIFLYMGVTSLNGIQ----------------FYERLHLLL 1143
SLC4A3/AE3|P48751 IQEVREQRVTGVLIA--SLVGLSIVMGAV-LRRIPLAVLFGIFLYMGVTSLSGIQ----------------LSQRLLLIL 1135
SLC4A4/NBCe1|Q9Y6R1 FLGVREQRVTGTLVF--ILTGLSVFMAPI-LKFIPMPVLYGVFLYMGVASLNGVQ----------------FMDRLKLLL 930
SLC4A5/NBCe2|Q9BY07 FLGVREQRVTGIIVF--ILTGISVFLAPI-LKCIPLPVLYGVFLYMGVASLNGIQMGTGGSEFKIQKKLTPFWERCKLFL 997
SLC4A7/NBCn1|Q9Y6M7 FLGIREQRVTGLMIF--ILMGLSVFMTSV-LKFIPMPVLYGVFLYMGVSSLKGIQ----------------LFDRIKLFG 1062
SLC4A8/NDCBE|Q2Y0W8 FLGIREQRVTGLMIF--VLMGCSVFMTAI-LKFIPMPVLYGIFLYMGVSSLQGIQ----------------FFDRLKLFG 932
SLC4A9/AE4|Q96Q91 FLGIREQRLTGLVVF--ILTGASIFLAPV-LKFIPMPVLYGIFLYMGVAALSSIQ----------------FTNRVKLLL 865
SLC4A10/NBCn2|Q6U841 FLGIREQRVTGLMIF--ILMGSSVFMTSI-LKFIPMPVLYGVFLYMGASSLKGIQ----------------FFDRIKLFW 963

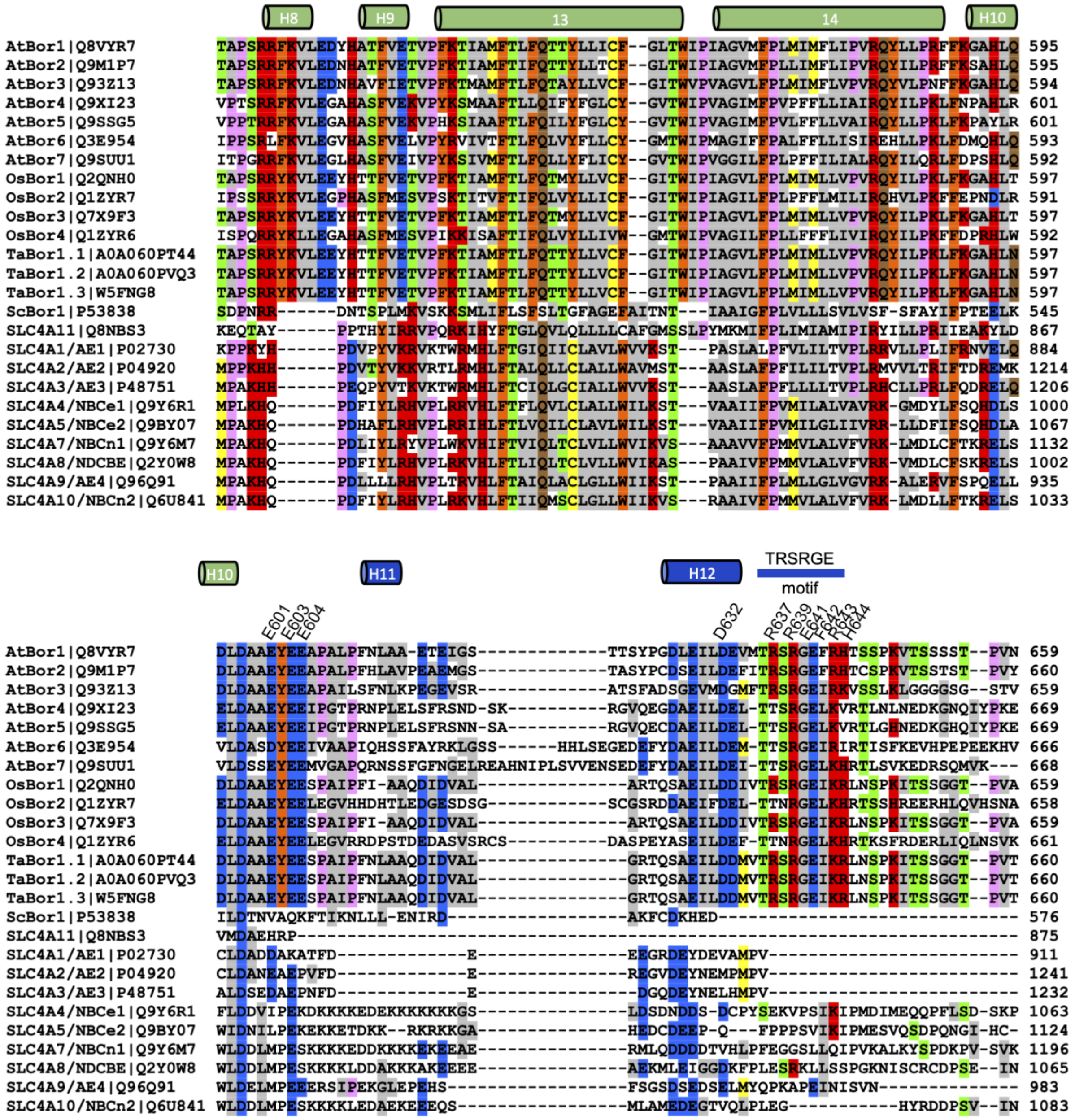

H8
H9
13
14
H10
AtBor1|Q8VYR7 TAPSRRFKVLEDYHATFVETVPFKTIAMFTLFQTTYLLICF--GLTWIPIAGVMFPLMIMFLIPVRQYLLPRFFKGAHLQ 595
AtBor2|Q9M1P7 TAPSRRFKVLEDNHATFVETVPFKTIAMFTIFQTTYLLTCF--GLTWIPIAGVMFPLLIMFLIPVRQYILPRFFKSAHLQ 595
AtBor3|Q93Z13 TAPSRRFKVLEDNHAVFIETVPFKTMAMFTLFQTAYLLVCF--GITWVPVAGVLFPLMIMFLVPVRQYVLPNFFKGAHLQ 594
AtBor4|Q9XI23 VPTSRRFKVLEGAHASFVEKVPYKSMAAFTLLQIFYFGLCY--GVTWIPVAGIMFPVPFFLLIAIRQYILPKLFNPAHLR 601
AtBor5|Q9SSG5 VPPTRRFKVLEGAHASFVEKVPHKSIAAFTLFQILYFGLCY--GVTWIPVAGIMFPVLFFLLVAIRQYLLPKLFKPAYLR 601
AtBor6|Q3E954 IPPSRLFKVLEGVHASFVELVPYRVIVTFTLFQLVYFLLCY--GMTWIPMAGIFFPALFFLLISIREHLLPKLFDMQHLQ 593
AtBor7|Q9SUU1 ITPGRRFKVLEGLHASFVEIVPYKSIVMFTLFQLLYFLICY--GVTWIPVGGILFPLPFFILIALRQYILQRLFDPSHLQ 592
OsBor1|Q2QNH0 TAPSRRYKVLEEYHTTFVETVPFKTIAMFTLFQTMYLLVCF--GITWIPIAGVLFPLMIMLLVPVRQYILPKLFKGAHLT 597
OsBor2|Q1ZYR7 IPSSRRYKVLEGPHASFMESVPSKTITVFTIFQLVYLLICF--GITWIPIAGILFPLPFFLMILIRQHVLPKFFEPNDLR 591
OsBor3|Q7X9F3 TAPSRRYKVLEEYHTTFVETVPFKTIAMFTLFQTMYLLVCF--GITWIPIAGVLFPLMIMLLVPVRQYILPKLFKGAHLT 597
OsBor4|Q1ZYR6 ISPQRRYKLLEGAHASFMESVPIKKISAFTIFQLVYLLIVW--GMTWIPVAGILFPLLFFFLIVIRQYILPKFFDPRHLW 592
TaBor1.1|A0A060PT44 TAPSRRYKVLEEYHTTFVETVPFKTIAMFTLFQTTYLLVCF--GITWIPIAGVLFPLMIMLLVPVRQYILPKLFKGAHLN 597
TaBor1.2|A0A060PVQ3 TAPSRRYKVLEEYHTTFVETVPFKTIAMFTLFQTTYLLVCF--GITWIPIAGVLFPLMIMLLVPVRQYILPKLFKGAHLN 597
TaBor1.3|W5FNG8 TAPSRRYKVLEEYHTTFVETVPFKTIAMFTLFQTTYLLVCF--GITWIPIAGVLFPLMIMLLVPVRQYILPKLFKGAHLN 597
ScBor1|P53838 SDPNRR------DNTSPLMKVSKKSMLIFLSFSLTGFAGEFAITNT---IAAIGFPLVLLLSVLVSF-SFAYIFPTEELK 545
SLC4A11|Q8NBS3 KEQTAY------PPTHYIRRVPQRKIHYFTGLQVLQLLLLCAFGMSSLPYMKMIFPLIMIAMIPIRYILLPRIIEAKYLD 867
SLC4A1/AE1|P02730 KPPKYH------PDVPYVKRVKTWRMHLFTGIQIICLAVLWVVKST---PASLALPFVLILTVPLRRVLLPLIFRNVELQ 884
SLC4A2/AE2|P04920 MPPKHH------PDVTYVKKVRTLRMHLFTALQLLCLALLWAVMST---AASLAFPFILILTVPLRMVVLTRIFTDREMK 1214
SLC4A3/AE3|P48751 MPAKHH------PEQPYVTKVKTWRMHLFTCIQLGCIALLWVVKST---AASLAFPFLLLLTVPLRHCLLPRLFQDRELQ 1206
SLC4A4/NBCe1|Q9Y6R1 MPLKHQ------PDFIYLRHVPLRRVHLFTFLQVLCLALLWILKST---VAAIIFPVMILALVAVRK-GMDYLFSQHDLS 1000
SLC4A5/NBCe2|Q9BY07 MPAKHQ------PDHAFLRHVPLRRIHLFTLVQILCLAVLWILKST---VAAIIFPVMILGLIIVRR-LLDFIFSQHDLA 1067
SLC4A7/NBCn1|Q9Y6M7 MPAKHQ------PDLIYLRYVPLWKVHIFTVIQLTCLVLLWVIKVS---AAAVVFPMMVLALVFVRK-LMDLCFTKRELS 1132
SLC4A8/NDCBE|Q2Y0W8 MPAKHQ------PDFIYLRHVPLRKVHLFTLIQLTCLVLLWVIKAS---PAAIVFPMMVLALVFVRK-VMDLCFSKRELS 1002
SLC4A9/AE4|Q96Q91 MPAKHQ------PDLLLLRHVPLTRVHLFTAIQLACLGLLWIIKST---PAAIIFPLMLLGLVGVRK-ALERVFSPQELL 935
SLC4A10/NBCn2|Q6U841 MPAKHQ------PDFIYLRHVPLRKVHLFTIIQMSCLGLLWIIKVS---RAAIVFPMMVLALVFVRK-LMDLLFTKRELS 1033
H10
H11
H12
TRSRGE
motif
E601
E603
E604
D632
R637
R639
E641
F642
R643
H644
AtBor1|Q8VYR7 DLDAAEYEEAPALPFNLAA-ETEIGS-------------TTSYPGDLEILDEVMTRSRGEFRHTSSPKVTSSSST--PVN 659
AtBor2|Q9M1P7 DLDAAEYEEAPALPFHLAVPEAEMGS-------------TASYPCDSEILDEFITRSRGEFRHTCSPKVTSSTST--PVY 660
AtBor3|Q93Z13 DLDAAEYEEAPAILSFNLKPEGEVSR-------------ATSFADSGEVMDGMFTRSRGEIRKVSSLKLGGGGSG--STV 659
AtBor4|Q9XI23 ELDAAEYEEIPGTPRNPLELSFRSND-SK----------RGVQEGDAEILDEL-TTSRGELKVRTLNLNEDKGNQIYPKE 669
AtBor5|Q9SSG5 ELDAAEYEEIPGTPRNPLELSFRSNN-SA----------RGVQECDAEILDEL-TTSRGELKVRTLGHNEDKGHQIYPKE 669
AtBor6|Q3E954 VLDASDYEEIVAAPIQHSSFAYRKLGSS------HHLSEGEDEFYDAEILDEM-TTSRGEIRIRTISFKEVHPEPEEKHV 666
AtBor7|Q9SUU1 VLDSSEYEEMVGAPQRNSSFGFNGELREAHNIPLSVVENSEDEFYDAEILDEI-TTSRGELKHRTLSVKEDRSQMVK--- 668
OsBor1|Q2QNH0 DLDAAEYEESPAIPFI-AAQDIDVAL---------------ARTQSAEILDDIVTRSRGEIKRLNSPKITSSGGT--PVA 659
OsBor2|Q1ZYR7 ELDAAEYEELEGVHHDHTLEDGESDSG------------SCGSRDDAEIFDEL-TTNRGELKHRTSSHREERHLQVHSNA 658
OsBor3|Q7X9F3 DLDAAEYEESPAIPFI-AAQDIDVAL---------------ARTQSAEILDDIVTRSRGEIKRLNSPKITSSGGT--PVA 659
OsBor4|Q1ZYR6 ELDAAEYEELEGVRRDPSTDEDASVSRCS----------DASPEYASEILDEF-TTNRGELKHRTKSFRDERLIQLNSVK 661
TaBor1.1|A0A060PT44 DLDAAEYEESPAIPFNLAAQDIDVAL---------------GRTQSAEILDDMVTRSRGEIKRLNSPKITSSGGT--PVT 660
TaBor1.2|A0A060PVQ3 DLDAAEYEESPAIPFNLAAQDIDVAL---------------GRTQSAEILDDMVTRSRGEIKRLNSPKITSSGGT--PVT 660
TaBor1.3|W5FNG8 DLDAAEYEESPAIPFNLAAQDIDVAL---------------GRTQSAEILDDMVTRSRGEIKRLNSPKITSSGGT--PVT 660
ScBor1|P53838 ILDTNVAQKFTIKNLLL-ENIRD------------------AKFCDKHED------------------------------ 576
SLC4A11|Q8NBS3 VMDAEHRP-- 875
SLC4A1/AE1|P02730 CLDADDAKATFD-------------E---------------EEGRDEYDEVAMPV------------------------- 911
SLC4A2/AE2|P04920 CLDANEAEPVFD-------------E---------------REGVDEYNEMPMPV------------------------- 1241
SLC4A3/AE3|P48751 ALDSEDAEPNFD-------------E----------------DGQDEYNELHMPV------------------------- 1232
SLC4A4/NBCe1|Q9Y6R1 FLDDVIPEKDKKKKEDEKKKKKKKGS---------------LDSDNDDS-DCPYSEKVPSIKIPMDIMEQQPFLSD-SKP 1063
SLC4A5/NBCe2|Q9BY07 WIDNILPEKEKKETDKK--RKRKKGA---------------HEDCDEEP-Q---FPPPSVIKIPMESVQSDPQNGI-HC- 1124
SLC4A7/NBCn1|Q9Y6M7 WLDDLMPESKKKKEDDKKKKEKEEAE---------------RMLQDDDDTVHLPFEGGSLLQIPVKALKYSPDKPV-SVK 1196
SLC4A8/NDCBE|Q2Y0W8 WLDDLMPESKKKKLDDAKKKAKEEEE---------------AEKMLEIGGDKFPLESRKLLSSPGKNISCRCDPSE--IN 1065
SLC4A9/AE4|Q96Q91 WLDELMPEEERSIPEKGLEPEHS------------------FSGSDSEDSELMYQPKAPEINISVN-------------- 983
SLC4A10/NBCn2|Q6U841 WLDDLMPESKKKKLEDAEKEEEQS-----------------MLAMEDEGTVQLPLEG-----------HYRDDPSV--IN 1083

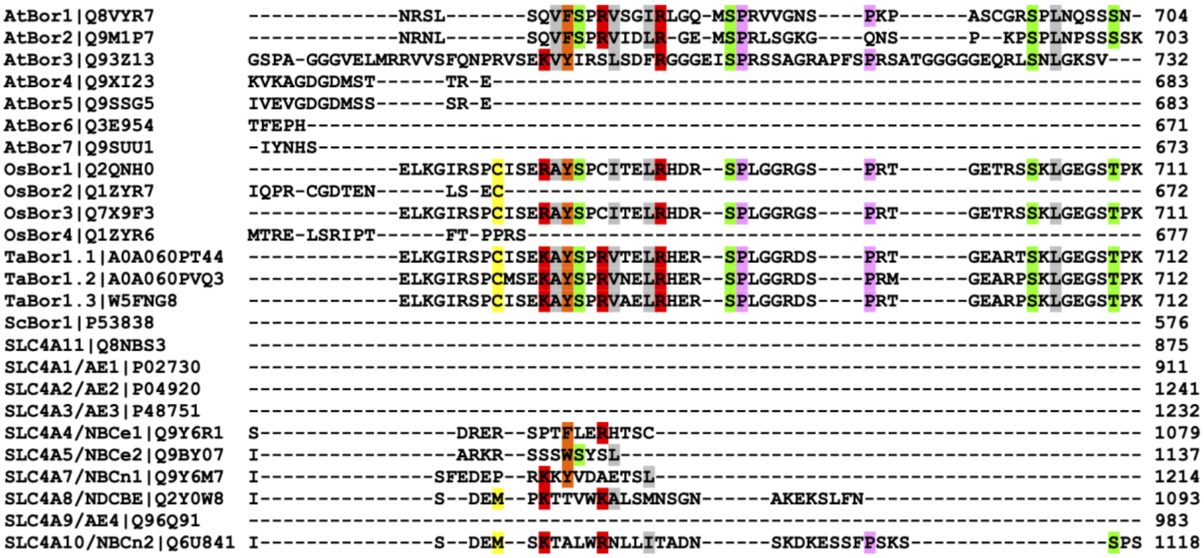

Supplementary Fig. 6 **Sequence alignment of *Arabidopsis thaliana*, rice, wheat borate transporters and human SLC4 transporters.** UniProt ID is shown after vertical slash. Secondary structural elements of AtBor1 are depicted above the sequence alignment. Dotted black lines represent unmodeled regions. Invariant and highly conserved residues are colored.

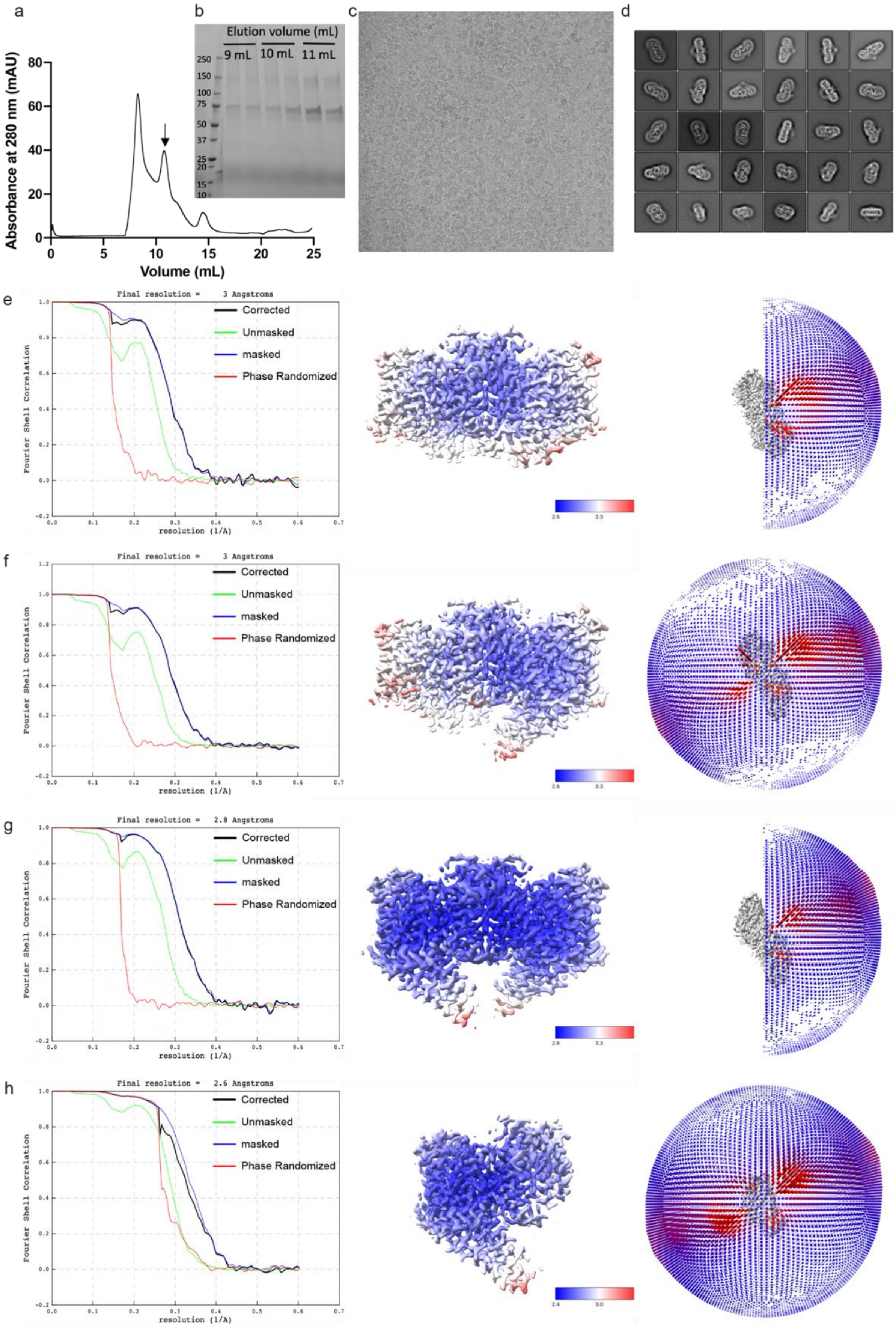

a
b
c
d
Absorbance at 280 nm (mAU)
Volume (mL)
Elution volume (mL)
9 mL
10 mL
11 mL
250
150
100
75
50
37
25
20
15
10
e
f
g
h
Final resolution = 3 Angstroms
Final resolution = 2.8 Angstroms
Final resolution = 2.6 Angstroms
Corrected
Unmasked
masked
Phase Randomized
Fourier Shell Correlation
resolution (1/A)
2.6
3.3
2.4
3.0

Supplementary Fig. 7 **Cryo-EM analysis of AtBor1$_{active}$ in 0.01% LMNG. a** Chromatographs of AtBor1$_{active}$ in 0.01% LMNG. The arrowed peak was collected for cryo-EM study. **b** The SDS-PAGE of the peak fractions. Monomeric AtBor1$_{active}$ protein was observed clearly on the SDS-PAGE between 75 to 50 KDa. **c** A representative electron micrograph of AtBor1$_{active}$ in 0.01% LMNG. **d** Representative two-dimensional class averages of the electron micrographs for AtBor1$_{active}$ in 0.01% LMNG. **e-h** Fourier Shell Correlation (FSC) curves (left), local resolution maps (middle), and particle orientation plots (right) of the 3D reconstruction of inward-facing dimer (**e**), hybrid inward-facing/occluded dimer (**f**), occluded dimer (**g**), and occluded protomer (**h**).

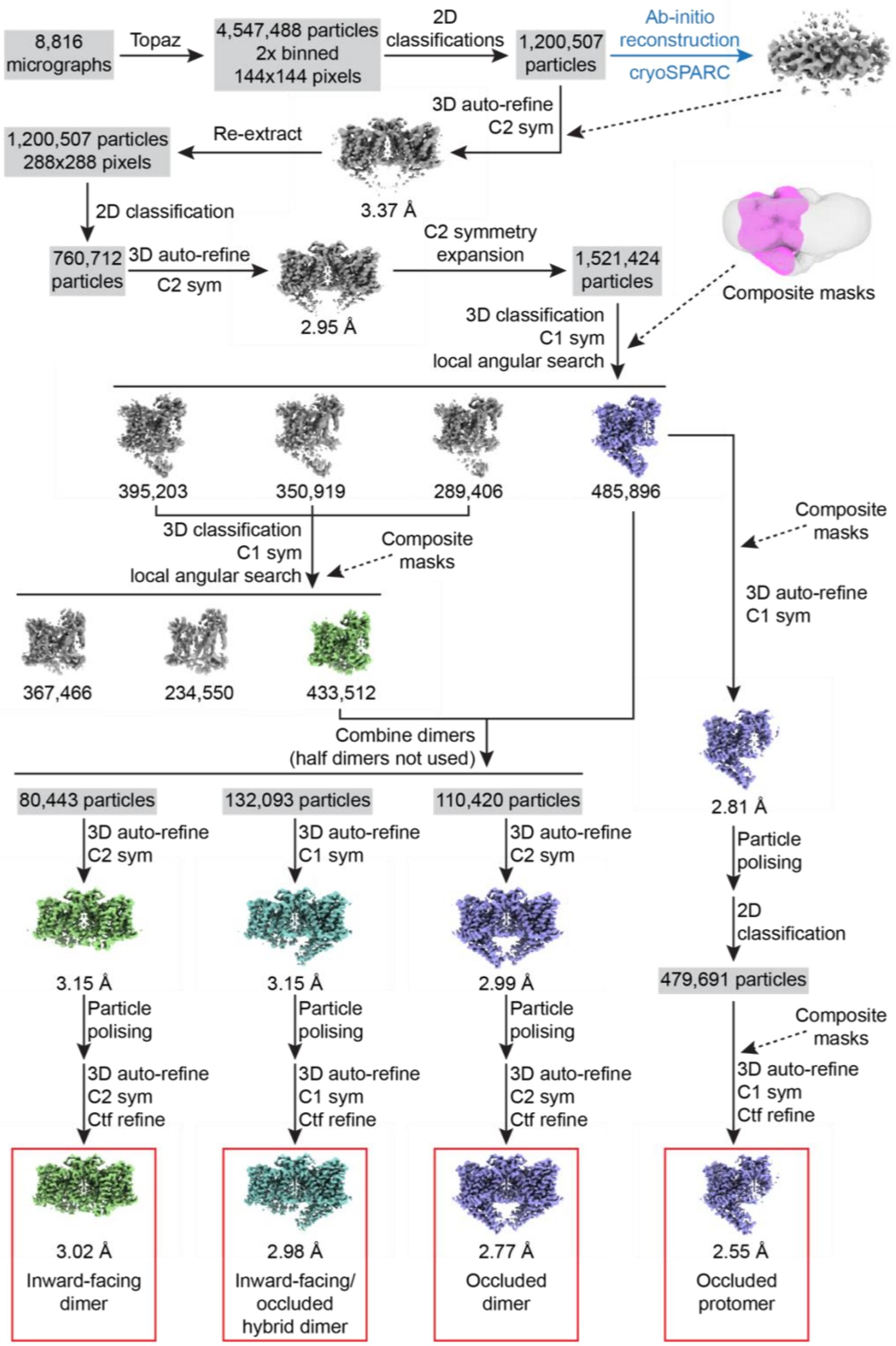

Supplementary Fig. 8 **Cryo-EM data processing workflow for AtBor1$_{\mathbf{active}}$.** The 3D reconstructions are shown without B-factor sharpening. The reconstructions in red boxes are used for atomic model building.

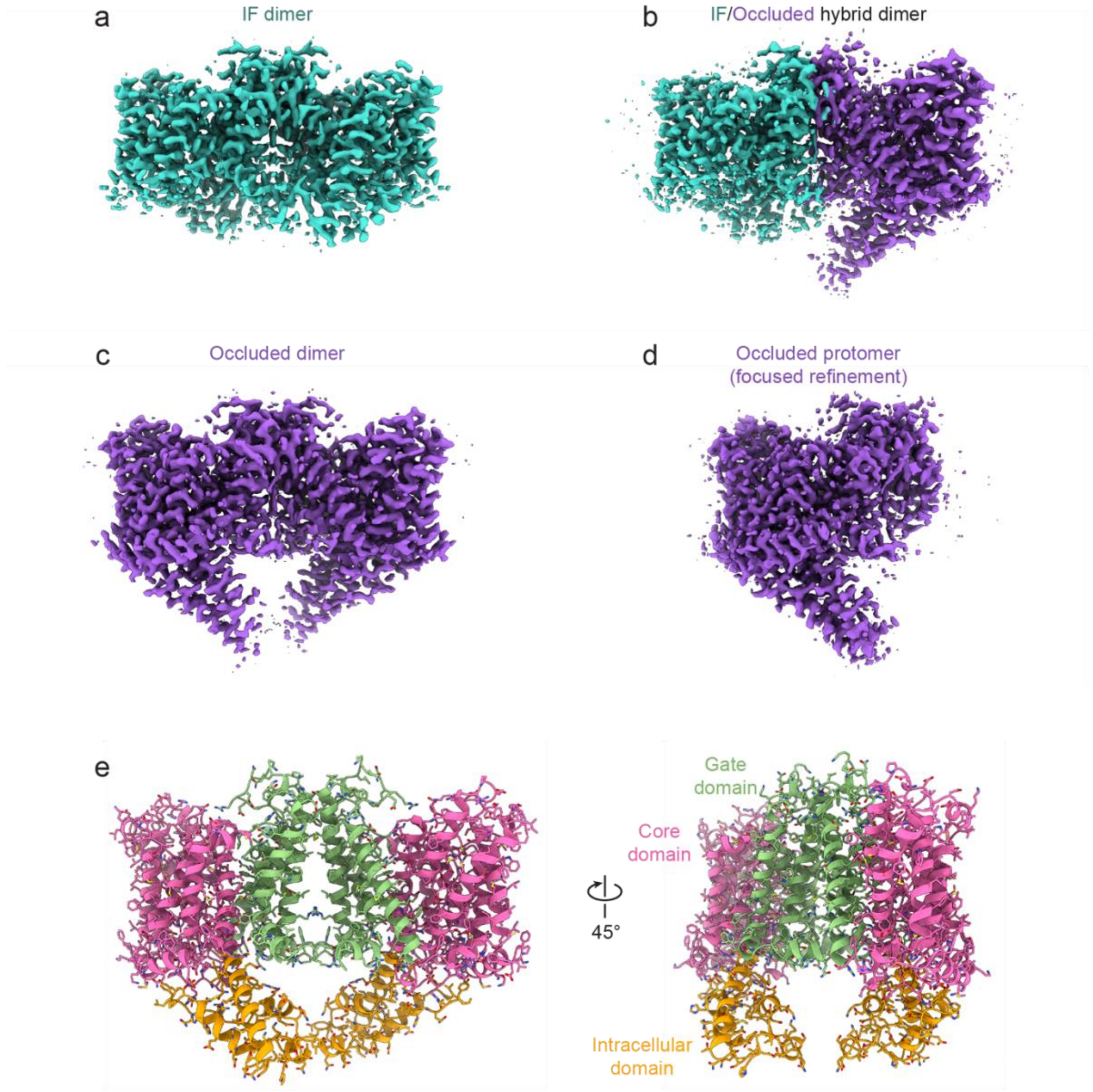

Supplementary Fig. 9 **Structures of active AtBor1. a-d,** Cryo-EM density maps of active AtBor1 IF dimer (**a**), IF/occluded hybrid dimer (**b**), occluded dimer (**c**) or occluded protomer (**d**). **e** The atomic model of active AtBor1 occluded dimer.

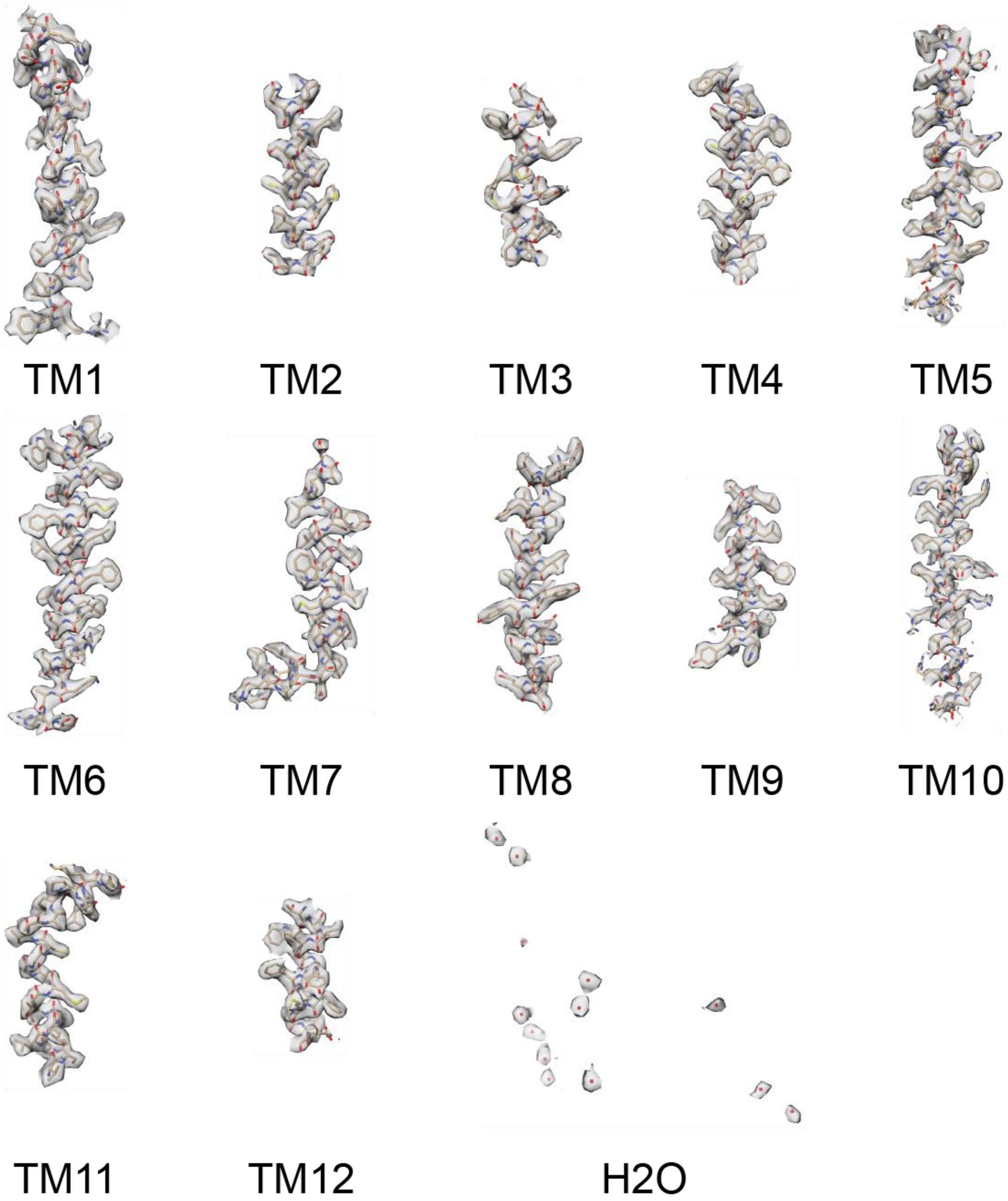

Supplementary Fig. 10 **Cryo-EM maps for representative regions of $AtBor1_{active}$.** The cryo-EM maps for the TM1-TM14 helices and the water molecules of $AtBor1_{active}$ in the occulded conformation.

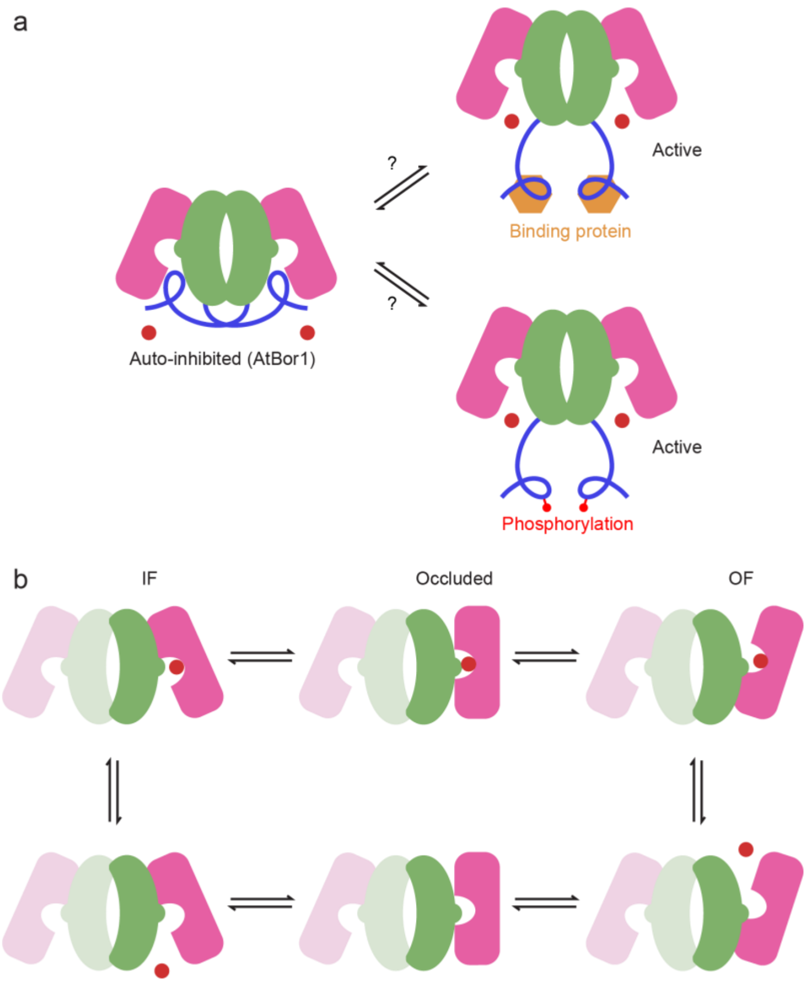

Supplementary Fig. 11 **The molecular basis of the elevator transport mechanism and the activity regulation for the SLC4 transporters. a** Proposed mechanisms for the release of autoinhibition. The Ct domain (blue) either interacts with an intracellular binding partner (yellow) or undergoes phosphorylation, leading to the release of the autoinhibitory domain. This conformational change allows substrates (red dots) to access the substrate binding pocket in the Core domain (pink) from the intracellular site. **b** Schematic representation of conformational transitions. In the inward-facing (IF) state, the substrate access to the substrate binding pocket in the Core domain (pink) from the intracellular site. Next, the Core domain moves upwards through the occluded state to the outward-facing (OF) state, allowing the substrate to be released from the transporter. The Core domain then moves downwards through the occluded conformation back to the inward-facing state in preparation for the next transport activity. The Gate domain (green) remains rigid during the process and the transport transition in one protomer is completely independent to the other protomer.

Supplementary Table 1 **Cryo-EM data collection, refinement, and validation statistics**

	WT AtBor1 protomer in lipid nanodiscs (EMD-41185) (PDB 8TEG)		WT AtBor1 dimer in lipid nanodiscs (EMD-41186) (PDB 8TEH)
**Data collection and processing**			
Magnification		165,000×	
Voltage (kV)		300	
Electron exposure (e–/Å^2)		55	
Defocus range (μm)		–0.8 to –2.5	
Pixel size (Å)		0.83	
Symmetry imposed	C1		C2
Initial particle images (no.)	2,485,991		2,485,991
Final particle images (no.)	853,837		1,012,376
Map resolution (Å)	2.15		2.30
FSC threshold	0.143		0.143
Map resolution range (Å)	2.1 – 3.0		2.2 – 5.2
**Refinement**			
Model resolution (Å)	2.1		2.3
FSC threshold	0.5		0.5
Map sharpening *B* factor (Å^2)	–48		–61
Model composition			
Non-hydrogen atoms	4,518		9,816
Protein residues	561		1,108
Ligands	0		24
Water	88		214
B factors (Å^2)			
Protein	27.98		28.28
Ligand	–		8.89
Water	27.17		26.87
R.m.s. deviations			
Bond lengths (Å)	0.007		0.003
Bond angles (°)	0.742		0.517
Validation			
MolProbity score	0.94		1.00
Clashscore	1.68		2.23
Poor rotamers (%)	1.05		0.43
Ramachandran plot			
Favored (%)	98.55		98.36
Allowed (%)	1.45		1.64
Disallowed (%)	0		0

Supplementary Table 2 **Cryo-EM data collection, refinement and validation statistics**

	$AtBor1_{active}$ occluded protomer (EMD-41188) (PDB 8TEJ)	$AtBor1_{active}$ occluded dimer (EMD-41190) (PDB 8TEL)	$AtBor1_{active}$ IF dimer (EMD-41191) (PDB 8TEM)	$AtBor1_{active}$ occluded/IF dimer (EMD-41192) (PDB 8TEN)
**Data collection and processing**				
Magnification	165,000×			
Voltage (kV)	300			
Electron exposure (e–/Å^2)	55			
Defocus range (μm)	–0.8 to –2.5			
Pixel size (Å)	0.83			
Symmetry imposed	C1	C2	C2	C1
Initial particle images (no.)	4,547,488	4,547,488	4,547,488	4,547,488
Final particle images (no.)	479,691	110,420	80,443	132,093
Map resolution (Å)	2.55	2.77	3.02	2.98
FSC threshold	0.143	0.143	0.143	0.143
Map resolution range (Å)	2.5 – 3.8	2.7 – 5.0	2.9 – 4.8	2.8 – 4.9
**Refinement**				
Model resolution (Å)	2.6	2.8	3.1	3.0
FSC threshold	0.5	0.5	0.5	0.5
Map sharpening *B* factor (Å^2)	–62	–58	–62	–60
Model composition				
Non-hydrogen atoms	3,925	7,806	7,992	7,872
Protein residues	494	988	1,010	996
Ligands	0	0	0	0
Water	22	0	0	0
B factors (Å^2)				
Protein	42.88	47.89	55.41	54.35
Ligand	–	–	–	–
Water	36.29	–	–	–
R.m.s. deviations				
Bond lengths (Å)	0.003	0.002	0.003	0.003
Bond angles (°)	0.513	0.489	0.557	0.518
Validation				
MolProbity score	1.74	1.45	1.59	1.36
Clashscore	2.78	3.10	3.52	3.70
Poor rotamers (%)	2.87	1.20	1.87	1.07
Ramachandran plot				
Favored (%)	95.26	95.77	96.39	96.94
Allowed (%)	4.74	4.23	3.61	3.06
Disallowed (%)	0	0	0	0

Supplementary Table 3 **List of oligonucleotides used in AtBor1 mutagenesis**

Mutations	Forward Primer	Reverse Primer
T636A	GTC ATG GCT AGA TCT AGA GGC GAG TTC AGA	AGA TCT AGC CAT GAC TTC GTC CAA GAT CTC
R637A	ATG ACC GCT TCT AGA GGC GAG TTC AGA CAC	TCT AGA AGC GGT CAT GAC TTC GTC CAA GAT
S638A	ACC AGA GCT AGA GGC GAG TTC AGA CAC	GCC TCT AGC TCT GGT CAT GAC TTC GTC
G640A	TCT AGA GCT GAG TTC AGA CAC ACC TCT TCC	GAA CTC AGC TCT AGA TCT GGT CAT GAC TTC
R639A	AGA TCT GCT GGC GAG TTC AGA CAC ACC	CTC GCC AGC AGA TCT GGT CAT GAC TTC GTC
R639K	AGA TCT AAG GGC GAG TTC AGA CAC ACC	CTC GCC CTT AGA TCT GGT CAT GAC TTC GTC
R639E	CAG ATC TGA GGG CGA GTT CAG ACA CAC C	TCGCCCTCAGATCTGGTCATGACTTCGTCC
E641A	AGA GGC GCT TTC AGA CAC ACC TCT TCC	TCT GAA AGC GCC TCT AGA TCT GGT CAT GAC
E641Q	AGA GGC CAG TTC AGA CAC ACC TCT TCC	TCT GAA CTG GCC TCT AGATCTGGTCATGAC
R643A	CGAGTTCGCTCACACCTCTTCCCCAAAGG	GTGTGAGCGAACTCGCCTCTAGATCTGG
H644A	GTTCAGAGCTACCTCTTCCCCAAAGGTTACC	GAGGTAGCTCTGAACTCGCCTCTAGATCTGG
R637E/E641R/R643E	G TCT AGA GGC AGA TTC GAG CAC ACC TCT TCC CCA AAG G	A ATC TGC CTC TAG ACT CGG TCA TGA CTT CGT CCA AGA TCT C
AtBor1/Bor4C	CTAGAGGCGAGTTGAAGGTTAGAACCTCTTCCCCAAAGGTTACC	TCAACTCGCCTCTAGAGGTGGTGACTTCGTCCAAGATCTCCAAATCG
Δ627-704	TGA TAA CTC GAG TCA TGT AAT TAG	GCC TGG GTA AGA GGT AGT AGA ACC
Δ627-641	TTC AGA CAC ACC TCT TCC CCA AAG	GCC TGG GTA AGA GGT AGT AGA ACC
Δ627-642	AGA CAC ACC TCT TCC CCA AAG GTT ACC	GCC TGG GTA AGA GGT AGT AGA ACC
Δ627-643	CAC ACC TCT TCC CCA AAG GTT ACC	GCC TGG GTA AGA GGT AGT AGA ACC
Δ627-644	ACC TCT TCC CCA AAG GTT ACC	GCC TGG GTA AGA GGT AGT AGA ACC
Δ627-645	TCT TCC CCA AAG GTT ACC TCT TCT TCC	GCC TGG GTA AGA GGT AGT AGA ACC
Δ638-641	TTC AGA CAC ACC TCT TCC CCA AAG	TCT GGT CAT GAC TTC GTC CAA GAT CTC
Δ636-641	TTC AGA CAC ACC TCT TCC CCA AAG	CAT GAC TTC GTC CAA GAT CTC CAA ATC
Δ651-704	CAAAGGTTTGATAACTCGAGTCATGTAATTAGT	GTTATCAAACCTTTGGGGAAGAGGTGT
R637E/E641R/R643E/T410V	ATG CAA GTC CCA TTG GTT TAC CAG CAG CCA	CAATGGGACTTGCATGTGGTGGTAAGCTTC
