# Supplementary material for: The Bor1 elevator transport cycle is subject to autoinhibition and activation
Source: Nat Commun. 2024 Oct 22;15:9090. doi: 10.1038/s41467-024-53411-1 (PMC11494103; doi:10.1038/s41467-024-53411-1)
Supplement: Supplementary file 2 — Description of Additional Supplementary Files [file 41467_2024_53411_MOESM2_ESM.pdf]

Description of Additional Supplementary Files

File Name: Supplementary Movie 1

Description: Spin movie showing interactions between the AID (blue) and transmembrane domains (core domain, pink; gate domain, green). Hydrogen bonds are shown as dashed lines.

File Name: Supplementary Movie 2

Description: Elevator transport mechanism in SLC4 transporter family, showing conformational changes between inward-facing AtBor1 and occluded AtBor1.

File Name: Supplementary Movie 3

Description: Elevator transport mechanism in SLC4 transporter family, showing conformational changes between inward-facing AtBor1, occluded AtBor1, and outward-facing human AE1 (PDB 7UZ3). Substrate coordination site is colored cyan.

File Name: Supplementary Movie 4

Description: Movie of substrate coordination site and FIFM gate, showing conformational changes between inwardfacing and occluded AtBor1.
