# Supplementary material for: The Bor1 elevator transport cycle is subject to autoinhibition and activation
Source: Nat Commun. 2024 Oct 22;15:9090. doi: 10.1038/s41467-024-53411-1 (PMC11494103; doi:10.1038/s41467-024-53411-1)
Supplement: Supplementary file 7 — Reporting Summary [file 41467_2024_53411_MOESM7_ESM.pdf]

Corresponding author(s): Yan Jiang and Jiansen Jiang

Last updated by author(s): 26/09/2024

Reporting Summary

Nature Portfolio wishes to improve the reproducibility of the work that we publish. This form provides structure for consistency and transparency in reporting. For further information on Nature Portfolio policies, see our Editorial Policies and the Editorial Policy Checklist.

Statistics

For all statistical analyses, confirm that the following items are present in the figure legend, table legend, main text, or Methods section.

n/a	Confirmed	
☐	☒	The exact sample size (*n*) for each experimental group/condition, given as a discrete number and unit of measurement
☒	☐	A statement on whether measurements were taken from distinct samples or whether the same sample was measured repeatedly
☒	☐	The statistical test(s) used AND whether they are one- or two-sided<br>*Only common tests should be described solely by name; describe more complex techniques in the Methods section.*
☒	☐	A description of all covariates tested
☒	☐	A description of any assumptions or corrections, such as tests of normality and adjustment for multiple comparisons
☒	☐	A full description of the statistical parameters including central tendency (e.g. means) or other basic estimates (e.g. regression coefficient) AND variation (e.g. standard deviation) or associated estimates of uncertainty (e.g. confidence intervals)
☒	☐	For null hypothesis testing, the test statistic (e.g. F, t, r) with confidence intervals, effect sizes, degrees of freedom and P value noted<br>*Give P values as exact values whenever suitable.*
☒	☐	For Bayesian analysis, information on the choice of priors and Markov chain Monte Carlo settings
☒	☐	For hierarchical and complex designs, identification of the appropriate level for tests and full reporting of outcomes
☒	☐	Estimates of effect sizes (e.g. Cohen's d, Pearson's r), indicating how they were calculated

Our web collection on statistics for biologists contains articles on many of the points above.

Software and code

Policy information about availability of computer code

Data collection	Leginon 3.5
Data analysis	RELION 3.0, cryoSPARC v2, MotionCor2 1.4.2, CTFFIND 4.1.14, and Topaz v0.2.4

For manuscripts utilizing custom algorithms or software that are central to the research but not yet described in published literature, software must be made available to editors and reviewers. We strongly encourage code deposition in a community repository (e.g. GitHub). See the Nature Portfolio guidelines for submitting code & software for further information.

Data

Policy information about availability of data

All manuscripts must include a data availability statement. This statement should provide the following information, where applicable:

- Accession codes, unique identifiers, or web links for publicly available datasets
- A description of any restrictions on data availability
- For clinical datasets or third party data, please ensure that the statement adheres to our policy

The cryo-EM maps have been deposited in the Electron Microscopy Data Bank (EMDB) under accession codes EMD-41185 [https://www.ebi.ac.uk/emdb/EMD-41185] (AtBor1 protomer), EMD-41186 [https://www.ebi.ac.uk/pdbe/entry/emdb/EMD-41186] (AtBor1 dimer), EMD-41188 [https://www.ebi.ac.uk/pdbe/entry/emdb/EMD-41188] (AtBor1active occluded protomer), EMD-41190 [https://www.ebi.ac.uk/pdbe/entry/emdb/EMD-41190] (AtBor1active occluded dimer), EMD-41191 [https://www.ebi.ac.uk/pdbe/entry/emdb/EMD-41191] (AtBor1active IF dimer) and EMD-41192 [https://www.ebi.ac.uk/pdbe/entry/emdb/

EMD-41192] (AtBor1active occluded/IF dimer). Atomic model coordinates have been deposited in the Protein Data Bank (PDB) under accession codes 8TEG [https://doi.org/10.2210/pdb8TEG/pdb] (AtBor1 protomer), 8TEH [https://doi.org/10.2210/pdb8TEH/pdb] (AtBor1 dimer), 8TEJ [https://doi.org/10.2210/pdb8TEJ/pdb] (AtBor1active occluded protomer), 8TEL [https://doi.org/10.2210/pdb8TEL/pdb] (AtBor1active occluded dimer), 8TEM [https://doi.org/10.2210/pdb8TEM/pdb] (AtBor1active IF dimer), 8TEN [https://doi.org/10.2210/pdb8TEN/pdb] (AtBor1active occluded/IF dimer). The previously published structural model of AE1 used in this research is available from the PDB under accession code 7UZ3 [http://doi.org/10.2210/pdb7UZ3/pdb]. The source data underlying the protein purification chromatographs in Supplementary Figures 1a and 7a, the uncropped SDS-PAGE gels in Supplementary Figures 1b and 7b, and the uncropped images of the agar plates from the yeast complementation assay (Figures 2g, 4e, and Supplementary Figure 5) are provided in the Source Data file.

Research involving human participants, their data, or biological material

Policy information about studies with human participants or human data. See also policy information about sex, gender (identity/presentation), and sexual orientation and race, ethnicity and racism.

Reporting on sex and gender	N/A
Reporting on race, ethnicity, or other socially relevant groupings	N/A
Population characteristics	N/A
Recruitment	N/A
Ethics oversight	N/A

Note that full information on the approval of the study protocol must also be provided in the manuscript.

Field-specific reporting

Please select the one below that is the best fit for your research. If you are not sure, read the appropriate sections before making your selection.

☒ Life sciences ☐ Behavioural & social sciences ☐ Ecological, evolutionary & environmental sciences

For a reference copy of the document with all sections, see nature.com/documents/nr-reporting-summary-flat.pdf

Life sciences study design

All studies must disclose on these points even when the disclosure is negative.

Sample size	Sample sizes for cryo-EM (numbers of particles or micrographs) were chosen to assure reaching the highest possible resolution with a consideration of the performance of the electron microscope and the detector.
Data exclusions	Cryo-EM micrographs with a resolution worse than 4 angstroms as reported by CTFFIND4 were excluded. Cryo-EM images of protein particles that belong to poor 2D classes or 3D classes were excluded during data processing using RELION3.
Replication	Protein purification, cryo-EM sample preparation and yeast complementation assays were repeated >=2 times for each of the reported cryo-EM structures and yeast complementation assays.
Randomization	Randomization was done automatically by the cryo-EM data processing software for 3D refinement to calculate gold-standard Fourier shell correlation.
Blinding	The initial model of the cryo-EM structure was generated directly from the cryo-EM images without a prior knowledge of the structure or any human supervision.

Reporting for specific materials, systems and methods

We require information from authors about some types of materials, experimental systems and methods used in many studies. Here, indicate whether each material, system or method listed is relevant to your study. If you are not sure if a list item applies to your research, read the appropriate section before selecting a response.

Materials & experimental systems

n/a	Involved in the study
☒	☐ Antibodies
☐	☒ Eukaryotic cell lines
☒	☐ Palaeontology and archaeology
☒	☐ Animals and other organisms
☒	☐ Clinical data
☒	☐ Dual use research of concern
☒	☐ Plants

Methods

n/a	Involved in the study
☒	☐ ChIP-seq
☒	☐ Flow cytometry
☒	☐ MRI-based neuroimaging

Eukaryotic cell lines

Policy information about cell lines and Sex and Gender in Research

Cell line source(s)	S. cerevisiae yeast strain-INVSC1 (ThermoFisher; Catalog number: C81000) and S. cerevisiae Y01169 strain: MATa; ura3Δ0; leu2Δ0; his3Δ1; met15Δ0; YNL275w:kanMX4 (Euroscarf; ACCNO: Y01169).
Authentication	The cell lines were purchased for recombinant protein production and yeast complementary assay. Authentication was not conducted.
Mycoplasma contamination	No mycoplasma contamination was detected.
Commonly misidentified lines (See ICLAC register)	To our best knowledge, there are no commonly misidentified lines.

Plants

Seed stocks	N/A
Novel plant genotypes	N/A
Authentication	N/A
